# 1,3-Disilabicyclo[1.1.0]butane and η^2^‑Silene Nickel Complex Derived from a Bromosilene

**DOI:** 10.1021/acs.inorgchem.6c00544

**Published:** 2026-04-28

**Authors:** Shunya Honda, Shintaro Ishida, Takeaki Iwamoto

**Affiliations:** Department of Chemistry, Graduate School of Science, 13101 Tohoku University, Aoba-ku, Sendai 980-8578, Japan

## Abstract

The reactivity of halogen-substituted silenes (silicon–carbon doubly bonded compounds) is not well understood relative to alkenyl halides. Herein, we report the reductive dimerization and complexation of a stable bromosilene (**1**). The reaction of **1** with an equimolar amount of KC_8_ furnished a 1,3-disilabicyclo[1.1.0]­butane (*trans*-**3**) featuring a remarkably short bridgehead Si–Si bond [2.204(1) Å] and a very wide Si–Si–C_exocyclic_ angle of 173.0(1)°. Bulky alkyl groups on the bridgehead silicon atoms and less steric hindrance at the bridging carbon atoms lead to the short bridgehead bond. Bromosilene **1** reacted with Ni­(PMe_3_)_4_ to afford the corresponding η^2^-bromosilene nickel complex (**4**) as a minor product. Complex **4** exhibits a twisted square planar structure in the solid state. Structural and spectroscopic properties of **4** indicate considerable metallacyclic character.

## Graphical abstract

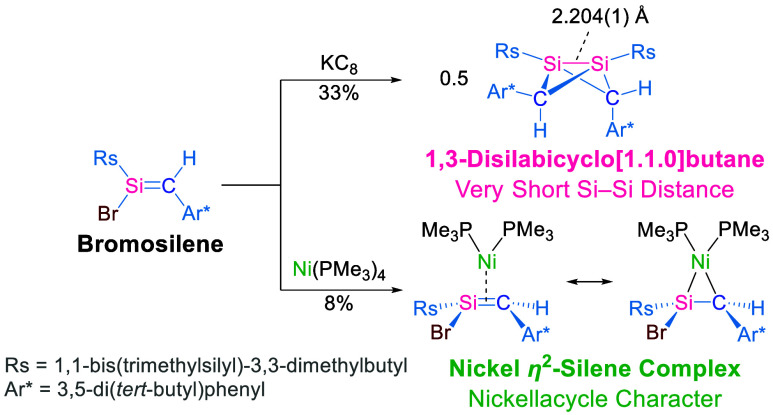

## Introduction

Silenes (Silicon–carbon doubly bonded compounds, R^1^R^2^Si=CR^3^R^4^) possess an intrinsically polarized Si^δ+^=C^δ−^ double bond due to the difference of the electronegativity between Si (1.90) and C (2.55) and a narrow π–π* gap owing to the poor 3p­(Si)–2p­(C) orbital overlap.^1–3^ Hence, the characteristic properties and reactivity of functionalized silenes receive broad attention.^4,5^ Among these, halogen-substituted silenes should enable us to access low-valent silicon-containing π-electron systems via functionalization of the halogen groups.^6–9^ However, their chemical transformations remain scarce relative to alkenyl halides. For instance, the treatment of NHC-coordinated trimethylsilyl­(chloro)­silene **A** with potassium amide furnishes the C=Si=Si=C cumulene derivative **B** via transmetalation and salt elimination (Scheme 1a).^7^ Bis-chlorosilene **C** reacts with B­(C_6_F_5_)_3_ to provide 1,4-disilabenzene **D** with elimination of chlorosilylene **E** (Scheme 1b).^8^ In this reaction, the two-electron oxidation of **C** to afford **F** is accompanied. Previously, we have reported the synthesis of stable bromosilene **1** and its substitution reactions using *tert*-butyllithium, lithium diphenylamide, and tris­(trimethylsilyl)­silylpotassium to deliver new silenes **2a**–**c** (Scheme 2a).^9^ Given that alkenyl halides, lighter congeners of halosilenes, are a versatile feedstock and useful reagents for numerous chemical transformations including coupling reactions,^10^ we decided to further examine Wurtz-coupling using alkaline metals and related reagents and Yamamoto-type homocoupling using Ni(0) complexes^11^ of **1** to obtain extended Si=C frameworks. Herein we report the reductive debromination of **1** with KC_8_ giving a 1,3-disilabicyclo[1.1.0]­butane *trans*-**3** and the complexation with Ni­(PMe_3_)_4_ furnishing an η^2^-silene nickel complex **4** (Scheme 2b).

**Scheme 1:**
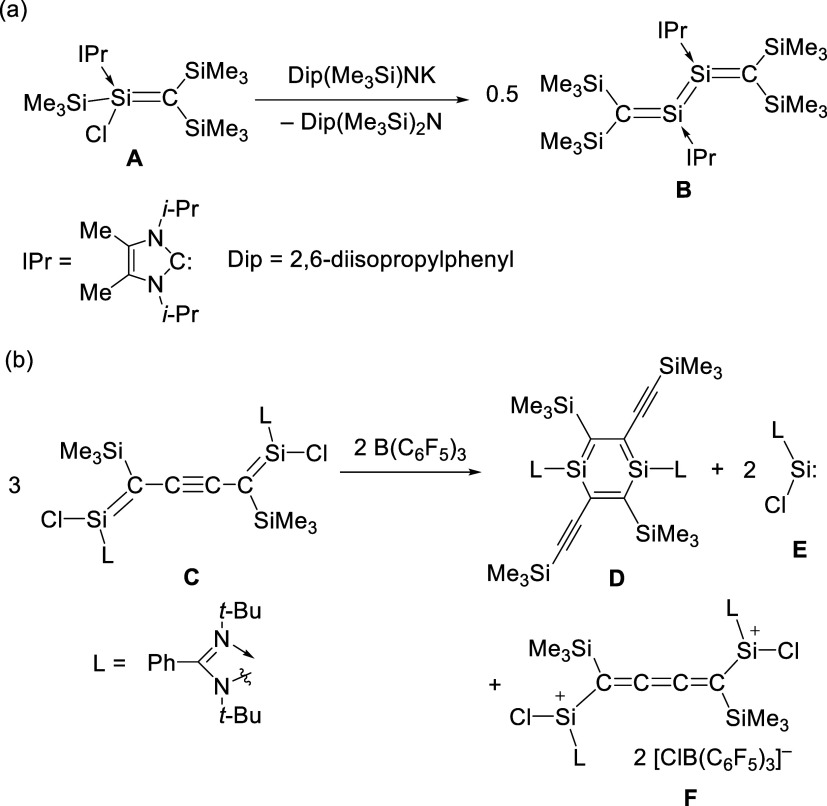
(a) Reaction of NHC-Coordinated Chlorosilene **A**.^7^ (b) Reaction of Bis-Chlorosilene **C** ^8^

**Scheme 2:**
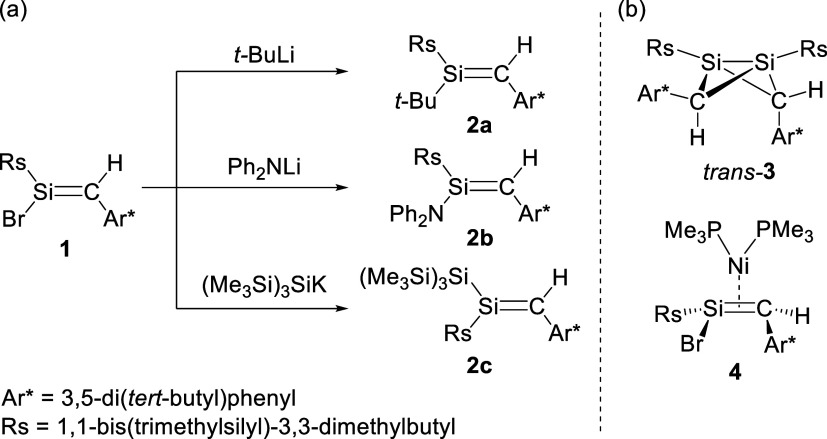
(a) Substitution Reactions of Bromosilene **1** with Nucleophiles to Afford **2a**–**c**.^9^ (b) 1,3-Disilabicyclo[1.1.0]­butane *trans*-**3** and η^2^-Silene Nickel Complex **4**

## Results and Discussion

### Reaction of Bromosilene 1 with KC_8_

Treatment of **1** with an equivalent amount of KC_8_ in diethyl ether at 10 °C for 3 h furnished 1,3-disilabicyclo[1.1.0]­butane *trans-* **3** instead of a homocoupling product, 2,3-disila-1,3-butadiene **3**′. *Trans*-**3** was isolated as a yellow crystalline solid in 33% yield by recrystallization from a saturated hexane solution at −25 °C (Scheme 3a). This is the first example of the formation of 1,3-disilabicyclo[1.1.0]­butane from halosilenes.^4f,12^ No other stereoisomers of *trans*-**3** were detected in the reaction mixture. Previously, we reported that 2,3-disila-1,3-butadiene **5a** thermally isomerizes into 1,3-disilabicyclo[1.1.0]­butane **6a** (Scheme 3b).^4f^ Computational studies suggested that the transformation of **5a** to **6a** proceeds via conrotatory ring closure. Consequently, it is reasonable to consider that the homocoupling of **1** provides **3**′ as an initial product, which undergoes the subsequent conrotatory cyclization to afford *trans*-**3** selectively (Scheme 3a).

**Scheme 3:**
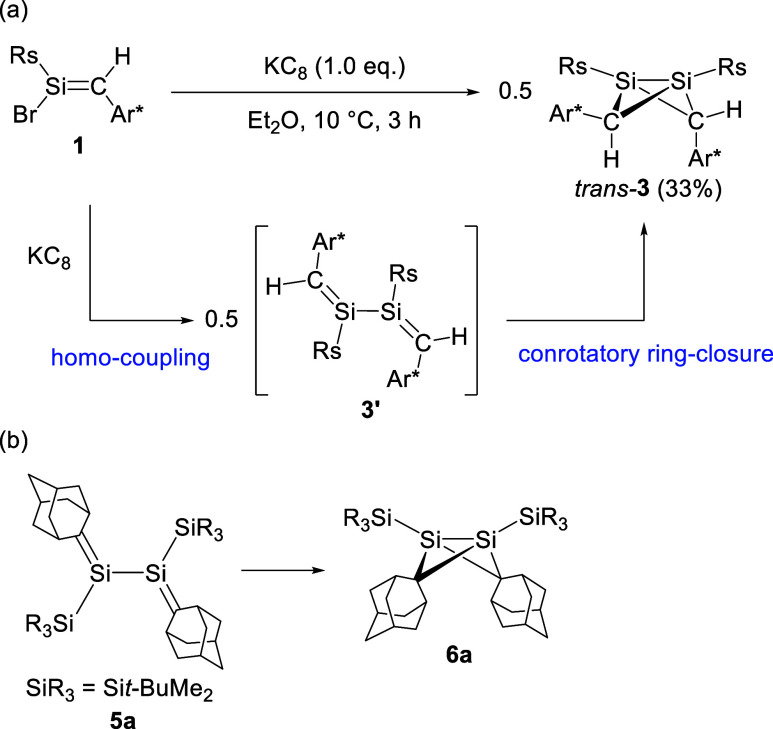
(a) Reaction of **1** with KC_8_; (b) Thermal Isomerization of **5a** to **6a** ^4f^

In the ^1^H NMR spectrum of *trans*-**3** in C_6_D_6_ at room temperature, diastereotopic methylene protons of 1,1-bis­(trimethylsilyl)-3,3-dimethylbutyl groups appeared as a set of two doublet signals. Two singlet signals due to bridging CAr*H units [Ar* = 3,5-di­(*tert*-butyl)­phenyl] appeared at 3.69 and 4.96 ppm. These spectroscopic characteristics confirm the *trans*-configuration of the bridging carbon atoms in the folded disilabicyclo[1.1.0]­butane skeleton. In the ^13^C­{^1^H} NMR spectrum, two tertiary signals and two quaternary signals due to the *para*- and *ipso*-positions of Ar* groups appeared in the aromatic region, indicating two chemically inequivalent Ar* groups. The signals of *ortho*-carbons are missing probably due to the signal broadening, which could result from the restricted ring rotation around the Ar*–C bond at room temperature.

The geometry around the bridgehead bond in bicyclo[1.1.0]­butanes involving heavy elements (heavy bicyclo[1.1.0]­butanes) such as *trans-* **3** has been discussed as an important issue because heavy bicyclo[1.1.0]­butanes are predicted to provide bond-stretch isomers which differ primarily in the distance between bridgehead atoms.^12,13^ The short-bond isomer (SBI) is characterized by a shorter bridgehead bond associated with a smaller hinge angle between the two three-membered rings, while the long-bond isomer (LBI) has the opposite trend. The molecular structure of *trans*-**3** in the solid state was determined by single crystal X-ray diffraction study (sc-XRD) as shown in Figure 1, and the obtained structural parameters represent marked characteristics of an SBI (Table 1). The bridgehead Si1–Si1* distance (*d*) of *trans*-**3** [2.204(1) Å] is much shorter than standard Si–Si bond lengths (2.36 Å)^14^ and the shortest value among hexaorganodisilanes (in the CSD, 215 entries in ConQuest Ver. 2025.3.1).

**Figure 1:**
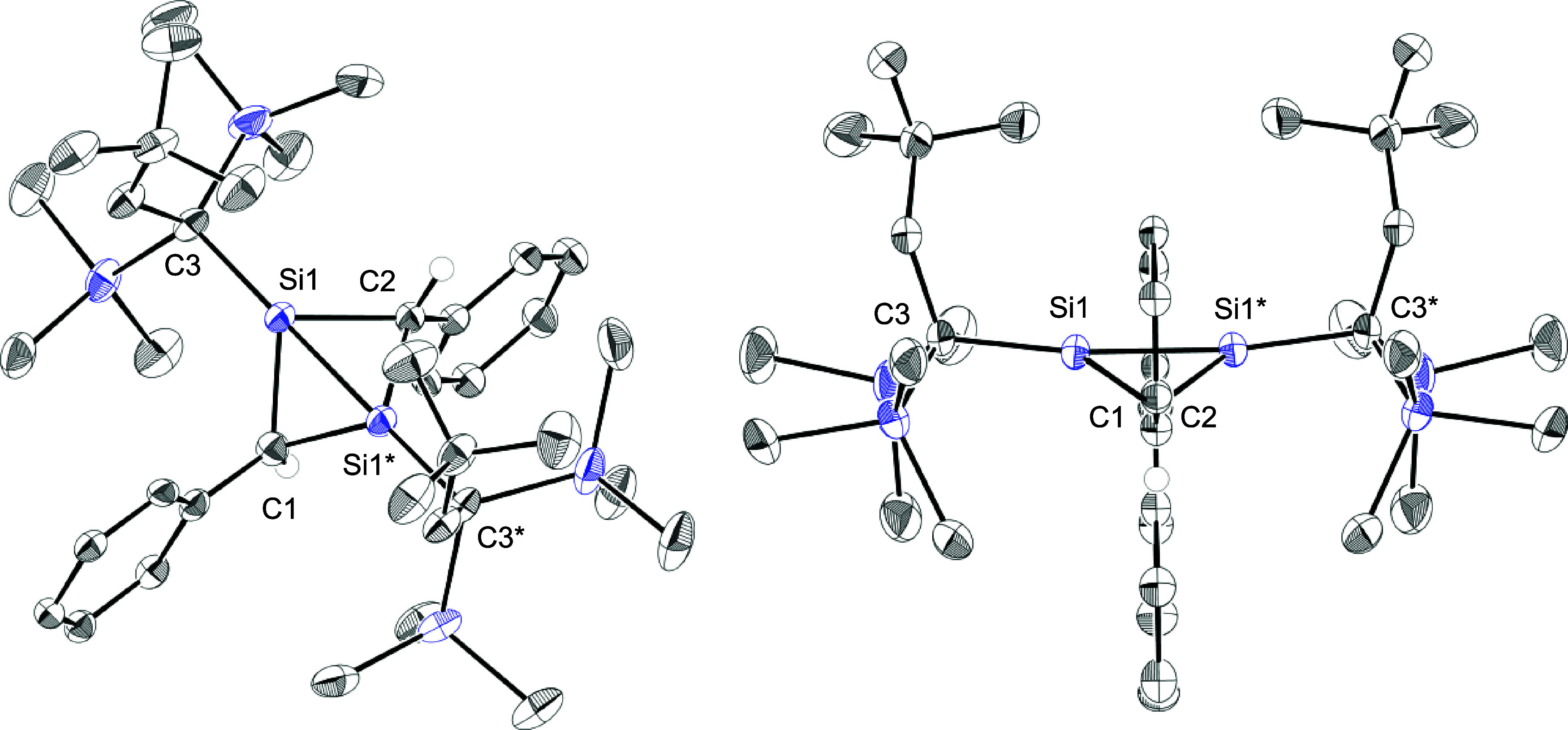
ORTEPs of *trans*-**3**. Hydrogen atoms except for those on the C1 and C2 atoms and *tert*-butyl groups on the aryl groups are omitted for clarity. Thermal ellipsoids are drawn at the 50% probability level. The 3,5-di­(*tert*-butyl)­phenyl (Ar*) rings and C1 and C2 atoms are located on the crystallographic mirror plane.

**Table 1:**
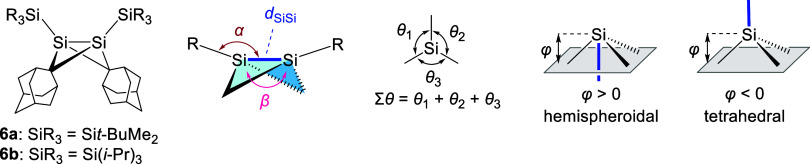
Selected Metric Parameters of *trans*-**3** and Related 1,3-Disilabicyclo[1.1.0]­butanes

compound	*d*_SiSi_/Å	α/°	β/°	Σθ^a^/°	φ^b^/Å	reference
*trans*-**3** ^c^	2.204(1)	173.0(1)	120.43	347.0	–0.387	this work
**6a** ^d^	2.3854(12)	119.19(5)	139.8(2)	338.95(14)	+0.548	^12a^
		119.78(5)		340.50(14)	+0.526	
	2.3867(13)	118.38(4)	140.2(2)	338.78(15)	+0.551	
		119.51(4)		340.11(15)	+0.531	
**6b**	2.2303(6)	144.96(2)	124.76(8)	359.96(5)	+0.022	^12b^
		144.21(2)		359.91(5)	+0.034	

a The angle sums around the bridgehead silicon atom except for the bridgehead bond.

b Distance of the central silicon atom and the reference plane, where the reference plane is defined by the three substituent atoms whose angle sum is closest to 360°. Negative φ indicates tetrahedral geometry at the silicon atom, while positive φ indicates hemispheroidal geometry.^15^

c A crystallographic mirror plane exists in the molecule.

d Two crystallographically independent molecules exist in the asymmetric unit.

It should be worth comparing the structures of 1,3-disilabicyclo[1.1.0]­butanes. We have reported 1,3-disilabicyclo[1.1.0]­butanes **6a** ^12a^ and **6b** ^12b^ (Table 1), and **6a** bearing *tert*-butyldimethylsilyl groups at the bridgehead positions exhibits an LBI character, while its triisopropylsilyl derivative **6b** is an SBI. 1,3-Disilabicyclo[1.1.0]­butanes having bulky substituents at the bridgehead positions tend to prefer the SBI.^12b^ The unusually short Si–Si bond in *trans*-**3** could arise mainly from steric effect imposed by the substituents and the bicyclic skeleton. The bridgehead Si1–Si1* bond lengths of 1,3-disilabicyclo[1.1.0]­butanes increase in the order: *trans*-**3** [2.204(1) Å] < **6b** [2.2303(6) Å] < **6a** [2.3854(12) and 2.3867(13) Å]. The hinge angles between two CSi_2_ three-membered rings (β) increase in the same order: *trans*-**3** [120.43°] < **6b** [124.76(8)°] < **6a** [144.1(1)°]. The short bridgehead bond and small β values are characteristic of the SBI and are consistent with the increasing bulkiness of the bridgehead substituents [*t*-BuCH_2_(Me_3_Si)_2_C (Rs) > *i*-Pr_3_Si > *t*-BuMe_2_Si]. In *trans*-**3**, the smaller bridging CHAr* group also allows a narrower β. The Rs–Si–Si angle (α) of *trans*-**3** [173.0(1)°] is much wider than the corresponding angles of **6a** [118.34(4)–119.78(5)°] and **6b** [144.21(2)° and 144.96(2)°]. A large α is typical of the SBI in heavy bicyclo[1.1.0]­butanes and would be enhanced by steric repulsion between the bulky Rs groups on the bridgehead silicon atoms. The geometry around the bridgehead silicon atoms can be categorized by (1) the angle sum at silicon excluding the bridgehead bond ∑θ­(Si), and (2) the hemispheroidality parameter φ.^15^ According to these parameters, **6a** [∑θ­(Si) = 339.59° and φ = +0.539 Å] and **6b** [∑θ­(Si) = 359.94° and φ = +0.028 Å] have hemispheroidal and trigonal pyramidal bridgehead silicon atoms, respectively, while the bridgehead silicon atoms in *trans*-**3** adopt a distorted tetrahedral geometry [∑θ­(Si) = 347.0° and φ = −0.387 Å]. These results indicate that the geometry of the bridgehead silicon atoms in the 1,3-disilabicyclo[1.1.0]­butanes is strongly influenced by the neighboring substituents.

UV–vis spectrum of *trans-* **3** in hexane exhibits an absorption band at 348 nm (band-I, ε 5200) as shown in Figure S13. We conducted TD-DFT calculations for the optimized structure of *trans*-**3** (**3**_**opt**_) at the M06–2X-D3/6–31+G­(d,p)//M06–2X-D3/6–31G­(d) level of theory to obtain insight into the electronic structure of *trans-* **3**. The calculated lowest energy transition of **3**_**opt**_ (347 nm) well reproduced the observed absorption band. Accordingly, the longest-wavelength absorption band of *trans*-**3** is assignable to the HOMO–LUMO transition. The HOMO and LUMO are bent σ- and σ*-orbitals at the bridgehead Si–Si bond (Figure 2). The calculated Mayer bond order (MBO)^16^ of the bridgehead silicon–silicon bond (0.75) is only slightly smaller than that of the Si–Si bond in hexamethyldisilane (0.86), which indicates a significant bonding interaction between the bridgehead silicon atoms. QTAIM analysis of **3**_**opt**_ also exhibits an outward-curved bond path between the bridgehead silicon atoms (Figure S14).^17^ The bond-path length (2.300 Å) is notably longer than the interatomic Si–Si distance (2.189 Å). The bond ellipticity at the bond critical point (BCP) on the Si–Si bond path is 0.182. The nonzero ellipticity value reflects a deviation from cylindrical symmetry of the electron density at the BCP, as observed for disilacyclopropane (ellipticity at the BCP of Si–Si bond: 0.359).^18^ Together, these computational features suggest a considerably bent banana-bond, as illustrated by the shape of the HOMO. Natural bond orbital (NBO) analysis provides information on the hybridization of the bridgehead Si–Si bond.^19^ The hybridization ratios of the bridgehead bonds are as follows: *trans*-**3** (s 16.79%, p 82.96%; sp^4.94^), **6a** (s 0.02%, p 99.78%; sp^99.99^), and **6b** (s 7.10%, p 92.69%; sp^13.05^). Bridgehead Si–Si bonds show high p-character in both the LBI and SBI structures, which is consistent with their large ∑θ­(Si) values and other bonding analyses. Among the SBI structures, *trans*-**3** has a larger s-character than **6b**. The difference may reflect the more electron-withdrawing alkyl substituent (Rs) relative to the silyl group and may also be relevant to the shorter Si–Si bond in *trans*-**3**.

**Figure 2:**
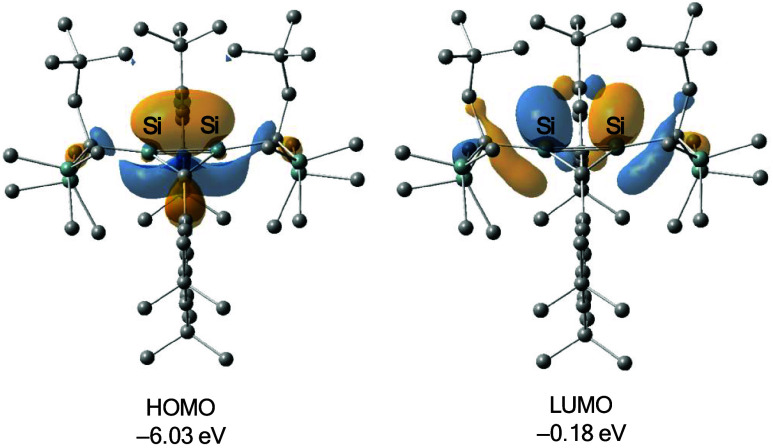
Frontier orbitals of **3**_**opt**_. Hydrogen atoms are omitted for clarity. Isosurface = 0.04.

### Reaction of Bromosilene with Ni­(PMe_3_)_4_

Treatment of **1** with Ni­(PMe_3_)_4_ in benzene at room temperature furnished a small amount of nickel η^2^-silene complex **4** together with an intractable mixture. Under these conditions, *trans*-**3** was not observed in the reaction mixture. After repeated recrystallizations, **4** was isolated in 8% yield as thermally unstable red crystals (Scheme 4). Because **1** decomposed in the presence of P­(*t*-Bu)_3_ to give a similar intractable mixture, decomposition of **1** induced by PMe_3_ released through ligand exchange is likely a major competing pathway in this reaction. Isolable group 10 metal η^2^-silene complexes are scarce, and structural information on this class of compounds is still very limited. Dinuclear nickel complex **7** and platinum complex **8** have been reported as isolable transition metal complexes of silenes and discussed in terms of their significant metallacyclic character.^4c,20,21^ Accordingly, we evaluate the bonding character of **4** in the context of the Dewar–Chatt–Duncanson model,^22^ using the upfield shifts in the NMR signals of the silene moiety and the structural changes upon complexation to access the relative contributions of metallacyclic and π-complex character. The methine proton on the silene ligand appeared at 1.90 ppm as a doublet (^3^ *J*_PH_ = 11.0 Hz), which was upfield-shifted by 4.22 ppm compared to that of free silene **1** (6.12 ppm). In the ^13^C and ^29^Si NMR spectra, the signal of the silene center was observed at 26.8 and 24.2 ppm, respectively, which are also considerably upfield-shifted upon complexation (^13^C and ^29^Si NMR chemical shifts of the silene moiety in **1** in C_6_D_6_: 100.9 and 85.2 ppm, respectively).^9^

**Scheme 4:**
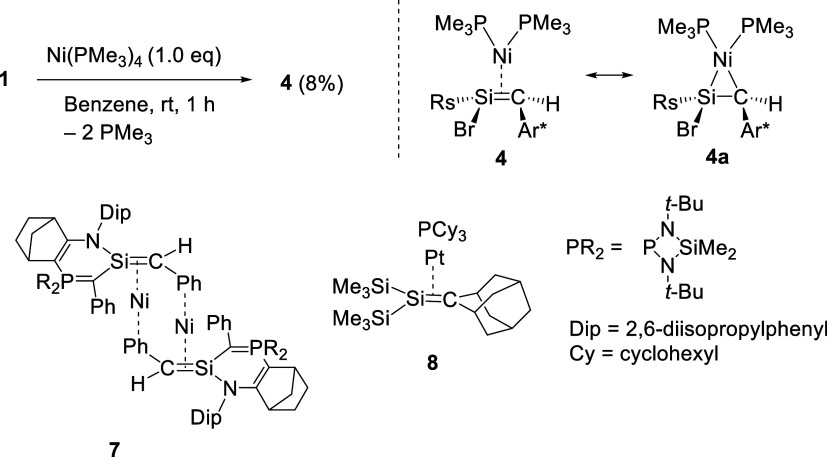
Reaction of **1** with Ni­(PMe_3_)_4_, Resonance Structures of **4**, and Previously Reported Silene-Nickel Complex **7** ^4c^ and Silene-Platinum Complex **8** ^20^

The structure of **4** in the solid state was determined by sc-XRD (Figure 3). Compound **4** was crystallized in the monoclinic *P*2_1_ space group, and two crystallographically independent but structurally similar molecules of **4** exist in the unit cell. Hereafter, only the molecule containing Ni1 is discussed. In complex **4**, the *Z*-configuration of the bromosilene moiety remains intact. The nickel atom adopts a slightly twisted square-planar coordination; the interplane angle between NiP_2_ and NiCSi planes is 14.8°. The steric repulsion among substituents on the silene and phosphines would cause the twisted structure. The bond length of the silene moiety (Si1–C1) [1.800(8) Å] is elongated by 4.9% from that of **1** [1.7157(15) Å] and falls into the range of the standard bond lengths of Si–C single bond (1.87 Å) and Si=C double bonds (1.70 Å). The Si=C bond length of **4** is comparable to those of **7** (1.825 Å) and **8** (1.838 Å), and the Si1–Ni1 and Ni1–C13 bond lengths [2.221(3) and 2.035(8) Å] are also similar to those of **7** [2.181 and 2.000 Å].^4c^ The Ni1–P2 (*trans*-position toward Si1) bond length of 2.206(3) Å is longer than that of Ni1–P1 (*cis*-position toward Si1) [2.156(2) Å] because of the trans influence of the σ-donating silyl ligand. The bromosilene unit of **4** diminished its planarity: the bent back angles, defined as an angle between an R_2_Si (R_2_C) plane and a C–Si bond, of the bromosilene moiety in **4** were 46.3° (Si1) and 21.2° (C1). Based on the observed upfield shifts in the NMR signals and the structural changes of the silene moiety, **4** has a significant metallacyclic character (**4a**), similar to that proposed for the previously reported η^2^-silene complexes **7** and **8**.^4c,20^

**Figure 3:**
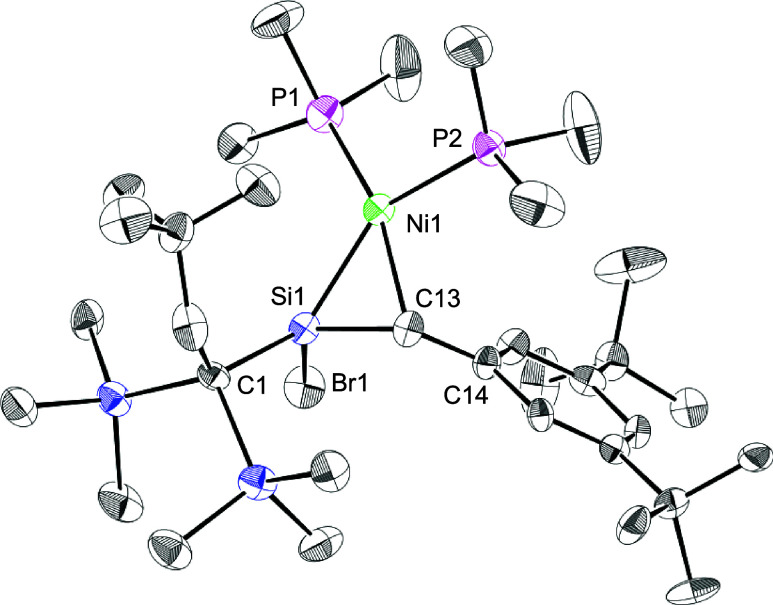
Molecular structures of **4**. Two crystallographically independent molecules exist in the unit cell, and one molecule is shown. Hydrogen atoms were omitted for clarity. Thermal ellipsoids are drawn at the 50% probability level. Selected bond lengths (Å): Si1–C13 1.800(8), Si1–Br1 2.269(2).

To obtain insight into the electronic structure of **4**, computational studies were carried out. The MBO analysis of **4**_**opt**_ at the M06–2X/B1 [B1 basis: SDD for Br and Ni atoms: 6–31G­(d) for H, C, Si, and P atoms] level of theory also confirmed the NiSiC metallacycle character. The MBOs of Si–Ni (0.70) and Ni–C13 (0.64) indicate the substantial bonding interaction between the nickel center and the silene moiety. The MBO of the Si–C13 bond (1.02) is significantly decreased from that of free bromosilene **1**_**opt**_ (1.68) at the same level of theory, demonstrating that the Si=C bond order is substantially reduced upon coordination to nickel, consistent with the structural changes observed upon complexation. Intrinsic bonding orbital (IBO) analysis provides a set of localized bonding orbitals without empirical inputs and further clarifies the bonding situations.^23–25^ The IBO analysis of **4**_**opt**_ at the PBE/def2-TZVP level of theory exhibits two n­(P), four d­(Ni), σ-donation, and π-backdonation orbitals (Figure 4). The σ-donation orbital is mainly localized on the carbon atom. The π-backdonation orbital is a bonding orbital between nickel and silicon atoms. The unsymmetric σ-donation and π-backdonation orbitals arise from the Si^δ+^=C ^δ−^ polarization. In the π-backdonation orbital, d-electron number on the nickel atom (1.251) is smaller than that of (Me_3_P)_2_Ni­(η^2^-ethylene), a typical Ni(0)-alkene complex (1.525, Figure S15). Hence, **4**_**opt**_ has a substantial metallacycle character compared to the π-complex (Me_3_P)_2_Ni­(η^2^-ethylene). The low-lying LUMO of **1** would enhance the π-backdonation, which is responsible for the metallacycle character of **4**.

**Figure 4:**
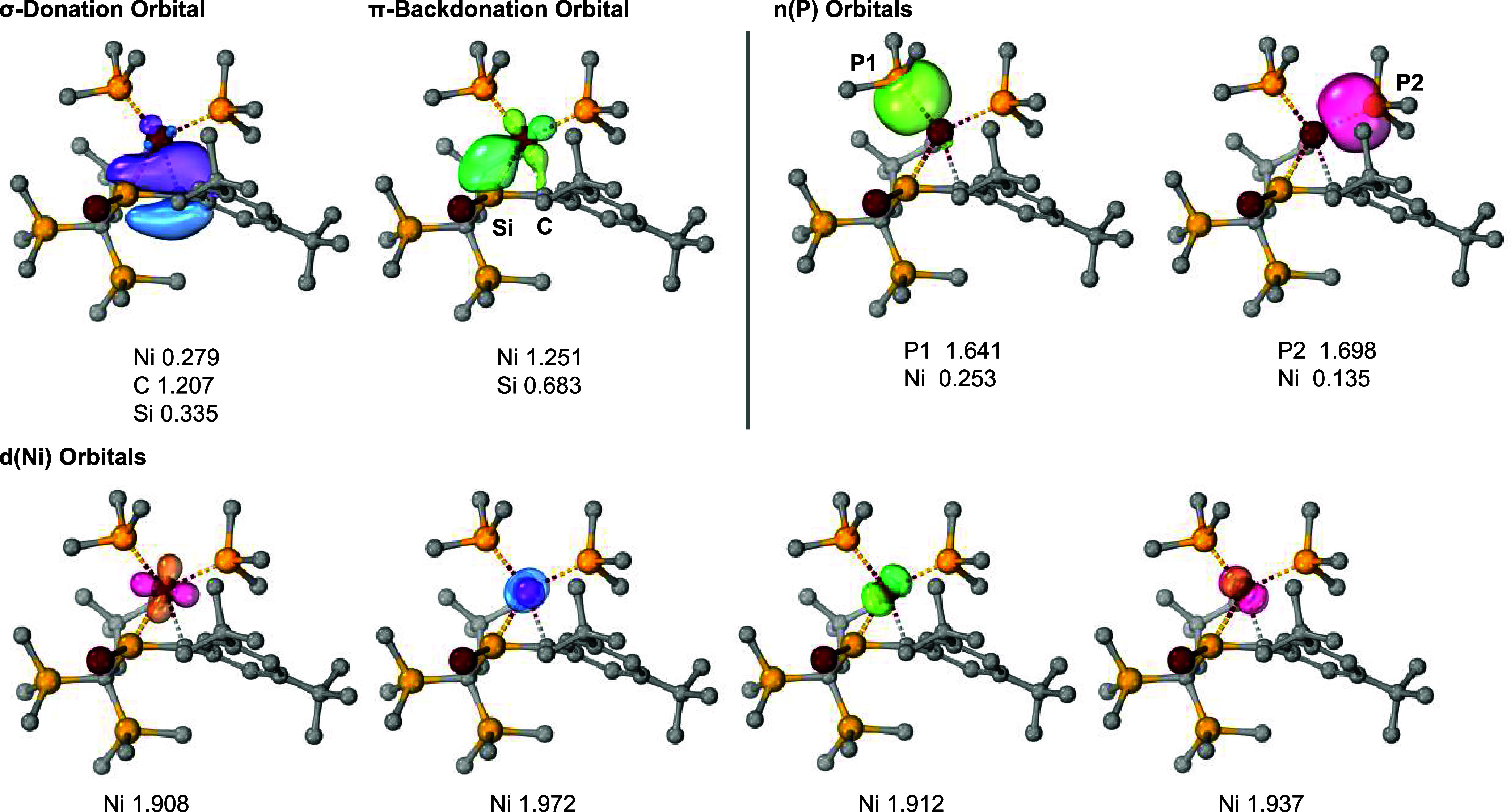
Selected IBOs of **4**_**opt**_ calculated at the PBE/def2-TZVP//M06–2X/B1 [B1 basis: SDD for Br and Ni atoms: 6–31G­(d) for H, C, Si, and P atoms] level of theory. Values indicate electron numbers on atoms in each IBO.

## Conclusion

Reductive debromination of bromosilene **1** with KC_8_ afforded a new 1,3-disilabicyclo[1.1.0]­butane (*trans*-**3**), which is a new route to 1,3-disilabicyclo[1.1.0]­butane from halosilene. *trans*-**3** features a large Si–Si–Rs bond angle [173.0(1)°] and a short Si–Si bond length [2.204(1) Å]. These findings suggest that a Si–Si single bond can be shortened by embedding it within a small bicyclic skeleton bearing bulky substituents at the bridgehead positions. In the reaction of **1** with Ni­(PMe_3_)_4_, a small amount of η^2^-silene nickel complex (**4**) was isolated. The spectroscopic, structural, and computational data for **4** indicate a considerable contribution from metallacycle **4a**.

### General Procedures

All reactions treating air-sensitive compounds were carried out under nitrogen atmosphere using a high-vacuum line and standard Schlenk techniques, or a glovebox as well as dry and oxygen-free solvents. ^1^H (500 MHz), ^13^C (126 MHz), ^29^Si (99 MHz), and ^31^P (202 MHz) NMR spectra were recorded on a Bruker Avance III 500 FT NMR spectrometer. ^1^H and ^13^C NMR chemical shifts were referenced to residual ^1^H and ^13^C of signals of benzene-*d*_6_ (^1^H δ 7.16 and ^13^C δ 128.0). ^29^Si NMR and ^31^P chemical shifts in benzene-*d*_6_ were referenced to external standards [Me_4_Si (δ 0.0) and 85% phosphoric acid (δ 0.0)]. Mass spectra were recorded on a Bruker Daltonics SolariX 9.4T and JEOL JMS-Q1050 spectrometers. UV–vis-NIR spectra were recorded in a JASCO V-770 spectrometer. X-ray analysis was carried out using a Bruker AXS APEX II CCD diffractometer. Measurement of melting point was measured on an SRS OptiMelt MPA100. Elemental analysis was performed with a J-SCIENCE LAB JM-11 at Research and Analytical Center for Giant Molecules, Graduate School of Science, Tohoku University.

### Materials

Diethyl ether (Et_2_O) and heptane were dried with LiAlH_4_ and distilled prior to use by using a vacuum line. Hexane and benzene were dried with a VAC-103991 type solvent purifier. Benzene-*d*_6_ was dried with molecular sieves 4Å. Materials unless otherwise noted are commercially available and used without further purification. KC_8_ ^26^ and bromosilene **1** ^9^ were prepared according to the literatures.

### Safety Statement

*Caution!* LiAlH_4_ and KC_8_ are extremely pyrophoric. They must be handled in a glovebox or using Schlenk lines.

### X-ray Crystallographic Analysis

Single crystals suitable for X-ray diffraction study were obtained by recrystallization from Et_2_O solution at −25 °C for *trans*-**3** and from heptane solution at −25 °C for **4**. The single crystals for data collection coated by Apiezon grease were mounted on a thin glass fiber and transferred to a cold nitrogen gas stream of the diffractometer. Diffraction data was collected by a Bruker AXS APEX II CCD diffractometer with graphite monochromated Mo–Kα radiation (0.71073 Å). An empirical absorption correction based on the multiple measurement of equivalent reflections was applied using the program SADABS^27^ and the structures were solved by direct methods and refined by full-matrix least-squares against *F* ^2^ using all data (SHELXL-2019).^28^ Molecular structure was analyzed by Yadokari-XG software.^29^

### Computational Study

All theoretical calculations were performed using Gaussian 09 ^30^ and GRRM 14 ^31–33^ programs. Geometry optimization, frequency analysis, and NBO analysis^19^ of **3**_**opt**_, **6a**, and **6b** were carried out at the M06–2X-D3/6–31G­(d) level of theory. Geometry optimization and frequency analysis of **4**_**opt**_ and **1**_opt_ were carried out at the M06–2X-D3/B1 (B1: SDD^34^ for Ni, Br, 6–31G­(d) for C, H, Si, P) level of theory. No imaginary frequencies were found in the optimized structures. Atomic coordinates of the optimized structures are listed in an xyz-format file (computational_study.xyz). Twenty excited states of **3**_**opt**_ calculated at the TD-M06–2*X*/6–31+G­(d,p) level of theory (Table S3). Mayer bond order (MBO) was calculated using Multiwfn software.^35^ Intrinsic bond orbital (IBOs) and Molecular graphs on QTAIM analysis are visualized by using IBOview^36^ and AIMALL^37^ programs.

### Synthesis

#### Reductive Debromination of Bromosilene 1 with KC_8_

In a test tube (20 mL) equipped with a magnetic stirring bar under argon atmosphere, bromosilene **1** (51 mg, 94 μmol) and potassium graphite (13 mg, 93 μmol) were placed. To the vial, Et_2_O (1 mL) was added. After stirring for 3 h at 10 °C, the volatiles of the reaction mixture were removed under reduced pressure. Then, hexane (ca. 5 mL) was added to the residue, and the insoluble material was filtered off using a PTFE filter, and the filtrate was concentrated under reduced pressure. The crude solid was recrystallized from hexane at −25 °C. Thus, obtained product was washed with cold heptane to yield *trans*-**3** (14 mg, 15 μmol) as pale-yellow crystals in 33% yield.

*trans-* **3**: pale-yellow crystals; ^1^H NMR (500 MHz, C_6_D_6_, 291 K, δ) 0.16 (s, 18H, SiMe_3_), 0.26 (s, 18H, SiMe_3_), 1.13 (s, 18H, *t-*Bu), 1.48 (s, 36H, *t-*Bu), 1.98 (d, 2H, CH_2_, *J* = 14.7 Hz), 2.04 (d, 2H, CH_2_, *J* = 14.7 Hz), 3.69 (s, 1H, Ar*CH), 4.96 (s, 1H, Ar*CH), 7.268 (s, 1H, *p*-CH), 7.270 (s, 1H, *p*-CH), 7.60 (very broad, 4H, *o*-CH); ^13^C­{^1^H} NMR (126 MHz, C_6_D_6_, 292 K, δ) 3.7 (SiMe_3_), 3.9 (SiMe_3_), 14.5 [*C*(SiMe_3_)_2_], 32.0 [C­(*C*H_3_)_3_], 32.1 [C­(*C*H_3_)_3_], 32.2 [CH_2_C­(*C*H_3_)_3_], 33.9 (CH_2_ *C*Me_3_), 35.1 (*C*Me_3_), 35.2 (*C*Me_3_), 41.4 (Ar*CH), 45.2 (CH_2_), 56.5 (Ar*CH), 117.3 (*p*-CH), 118.1 (*p*-CH), 145.5 (*ipso*-C), 148.0 (*ipso*-C), 150.4 (*m*-C), 150.7 (*m*-C); ^29^Si­{^1^H} NMR (99 MHz, C_6_D_6_, 291 K, δ) −14.9 (bridgehead Si), 2.1 (SiMe_3_), 3.3 (SiMe_3_); HRMS (APCI) *m*/*z* [M]^+^ calcd for C_54_H_102_Si_6_, 918.6592; Found, 918.6592; Anal. Calcd for C_54_H_102_Si_6_: C, 70.51; H,11.18%. Found: C, 70.61; H, 11.25%; UV–vis (hexane, 298 K) λ/nm (ε) 348 (5.61 × 10^3^). In the ^13^C­{^1^H} NMR spectrum, the signals for *ortho*-carbon atoms of 3,5-di­(*tert*-butyl)­phenyl groups were missing. As ^1^H NMR signals of *ortho*-protons were remarkably broadened, the reason for the absence of the corresponding ortho-carbon signals would also be broadening.

#### Reaction of 1 with Ni­(PMe_3_)_4_

In a Schlenk tube (20 mL) equipped with a magnetic stirring bar under argon atmosphere, Ni­(PMe_3_)_4_ (33 mg, 92 μmol) and benzene (1 mL) were placed. To the flask, a benzene solution (2 mL) of bromosilene **1** (50 mg, 93 μmol) was added as four portions. Then, the flask was carefully evacuated for 1 min to remove the resulting volatile trimethylphosphine. After stirring for 15 min under slightly reduced pressure and for 1 h under ambient pressure, the solution turned from yellow to orange. Volatiles were removed by reduced pressure, and hexane was added to the residue. The insoluble material was filtered off using a PTFE filter, and the filtrate was concentrated under reduced pressure. The crude product was recrystallized twice from hexane at −25 °C, and η^2^-bromosilene nickel complex **4** was obtained as red crystals in 8% yield (6 mg, 8 μmol).

**4**: red crystals; decomposed (>30 °C) before melting; ^1^H NMR (500 MHz, C_6_D_6_, 296 K, δ) 0.55 (s, 9H, SiMe_3_), 0.57 (s, 9H, SiMe_3_), 0.69 (d, 9H, PMe_3_, ^2^ *J*_PH_ = 6.0 Hz), 1.18 (d, 9H, PMe_3_, ^2^ *J*_PH_ = 6.5 Hz), 1.35 (s, 9H, CH_2_C*Me*_3_), 1.44 (s, 18H, *t-*Bu on Ar*), 1.90 (d, 1H, silene-CH, ^3^ *J*_PH_ = 11.0 Hz), 2.15 (d, 1H, C*H*_2_ *t*Bu, *J* = 14.8 Hz), 2.42 (d, 2H, C*H*_2_ *t*Bu, *J* = 14.8 Hz), 7.09 (s, 1H, *p*-CH), 7.45 (brs, 2H, *o*-CH); ^13^C­{^1^H} NMR (126 MHz, C_6_D_6_, 297 K, δ) 4.6 (SiMe_3_), 5.5 (SiMe_3_), 11.5 [d, *C*(SiMe_3_)_2_, ^3^ *J*_PC_ = 19.9 Hz], 16.2 (dd, PMe_3_, ^1^ *J*_PC_ = 17.2, ^3^ *J*_PC_ = 2.7 Hz), 19.6 (dd, PMe_3_, ^1^ *J*_PC_ = 22.2, ^3^ *J*_PC_ = 5.5 Hz), 26.8 (d, silene-CH, ^2^ *J*_PC_ = 3.9 Hz), 32.1 [C­(*C*H_3_)_3_], 33.4 [CH_2_C­(*C*H_3_)_3_], 33.9 (CH_2_ *C*Me_3_), 35.0 (C*C*Me_3_), 45.2 (CH_2_), 114.0 (CH), 122.0 (CH), 148.7 (C), 149.2 (C); ^29^Si­{^1^H} NMR (99 MHz, C_6_D_6_, 299 K, δ) 1.9 (SiMe_3_), 7.7 (SiMe_3_), 24.2 (dd, ^2^ *J*_PSi_ = 117.2, 24.0 Hz, silene-Si); ^31^P­{^1^H} NMR (202 MHz, C_6_D_6_, 296 K, δ) −20.9 (d, PMe_3_, ^2^ *J*_PP_ = 27.4 Hz), −16.3 (d, PMe_3_, ^2^ *J*_PP_ = 27.4 Hz); HRMS (APCI) *m*/*z* [M – PMe_3_ + 2O]^+^ calcd for C_30_H_61_BrNiO_2_PSi_3_, 705.2248; found; 705.2251, [M + 3O + H]^+^ calcd for C_33_H_70_BrNiO_3_P_2_Si_3_, 797.2639; found; 797.2643; Anal. Calcd for C_33_H_69_BrNiP_2_Si_3_: C, 52.80; H, 9.26%. Found: C, 52.75; H, 9.52%.

## Supplementary Material





## Notes

NMR and UV–vis spectra, crystallographic data, and computational study data (PDF)
Cartesian coordinates of all computed molecules (XYZ)
