# Supplementary material for: 1,3-Disilabicyclo[1.1.0]butane and η2‑Silene Nickel Complex Derived from a Bromosilene
Source: Inorg Chem. 2026 Apr 28;65(18):10087–95. doi: 10.1021/acs.inorgchem.6c00544 (PMC13169367; doi:10.1021/acs.inorgchem.6c00544)
Supplement: Supplementary file 1 [file ic6c00544_si_001.pdf]

Supporting Information

1,3-Disilabicyclo[1.1.0]butane and η^2-Silene Nickel Complex Derived from a Bromosilene

Shunya Honda, Shintaro Ishida,* and Takeaki Iwamoto*

Department of Chemistry, Graduate School of Science, Tohoku University, Sendai 980-8578, Japan

E-mail: takeaki.iwamoto@tohoku.ac.jp

Contents

1. NMR and UV-vis Spectra S2
2. X-ray Crystallographic Analysis Data S9
3. Computational Study S11

1. NMR and UV-vis Spectra

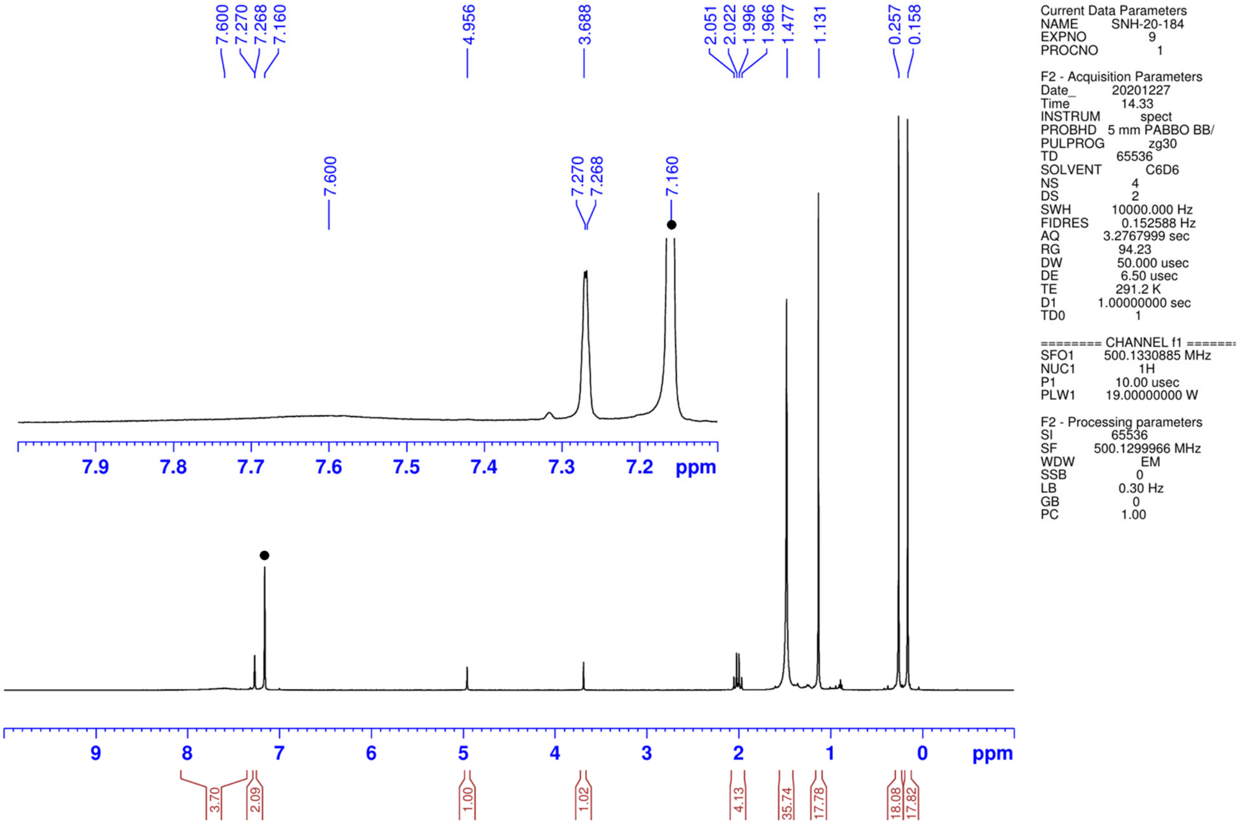

Figure S1. ^{1}H NMR spectrum of *trans*-**3** in C_6D_6 at 291 K (• = C_6D_5H).

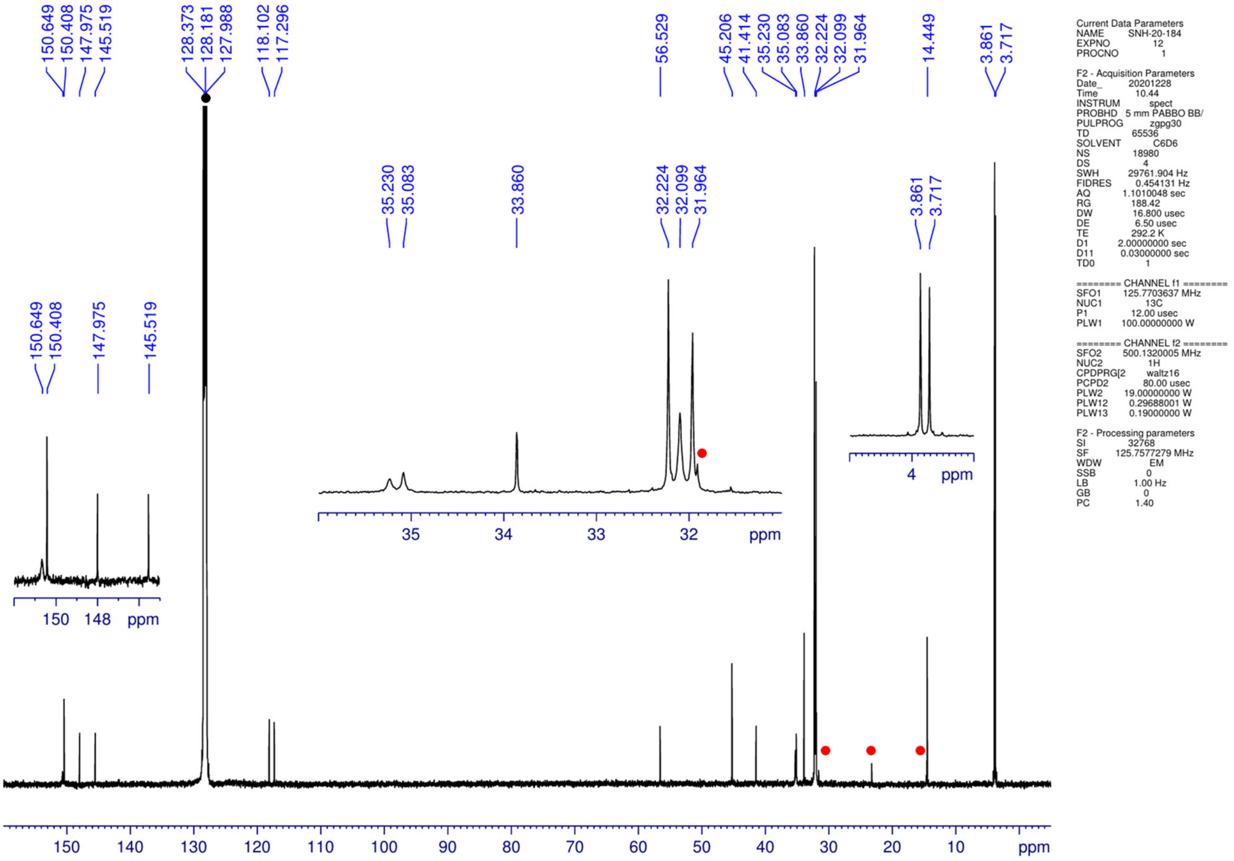

Figure S2. $^{13}C\{^{1}H\}$ NMR spectrum of *trans*-**3** in C_6D_6 at 292 K (• = C_6D_6 • = hexane).

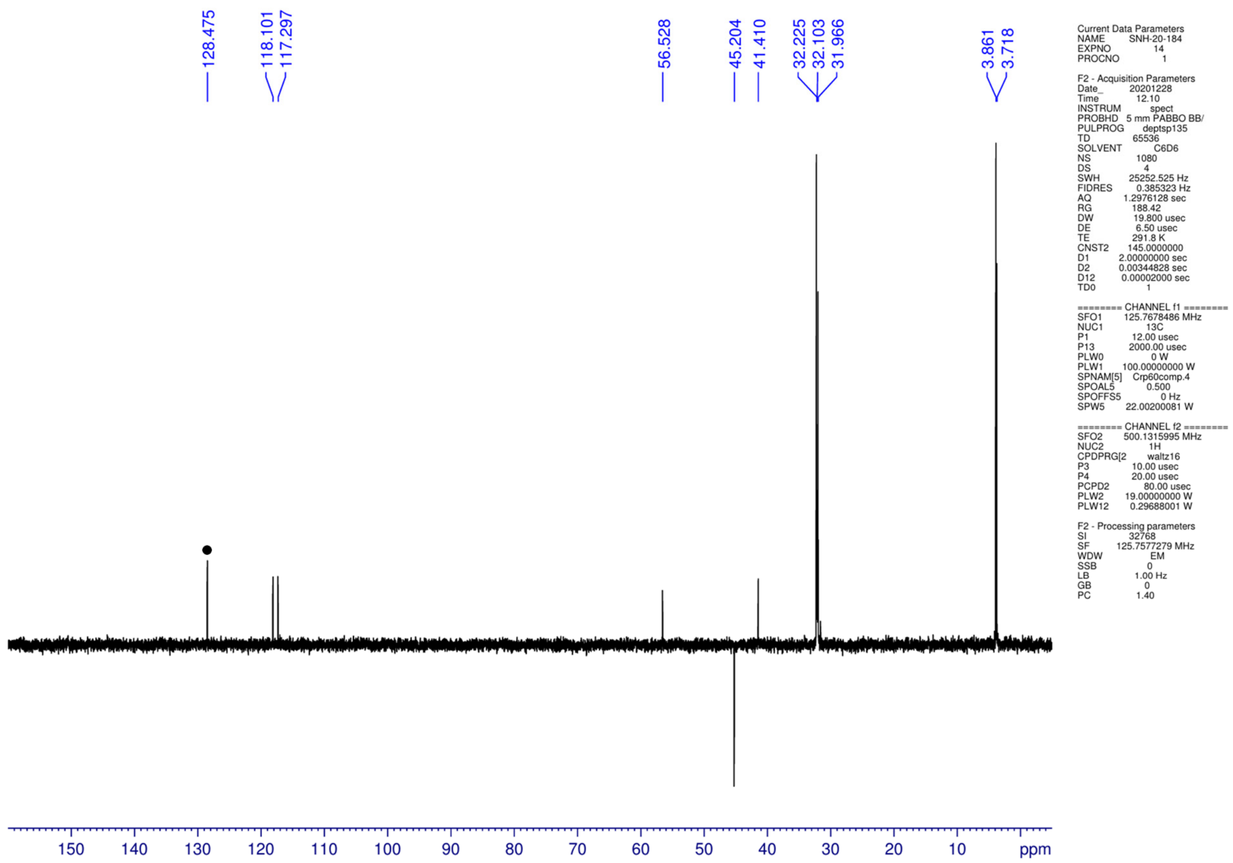

Figure S3. $^{13}C\{^1H\}$ dept135 NMR spectrum of *trans*-**3** in C_6D_6 at 291 K (•= C_6D_5H).

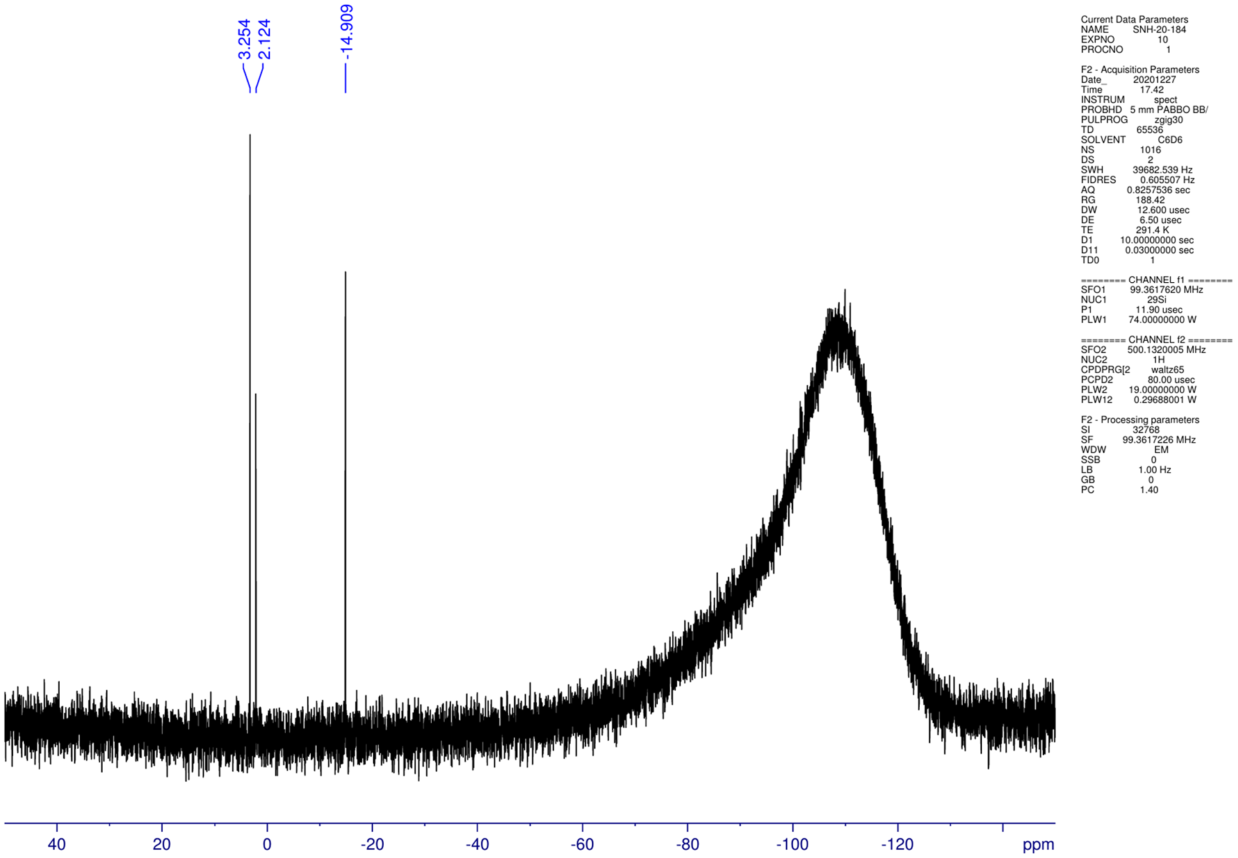

Figure S4. $^{29}Si\{^1H\}$ NMR spectrum of *trans*-**3** in C_6D_6 at 291 K.

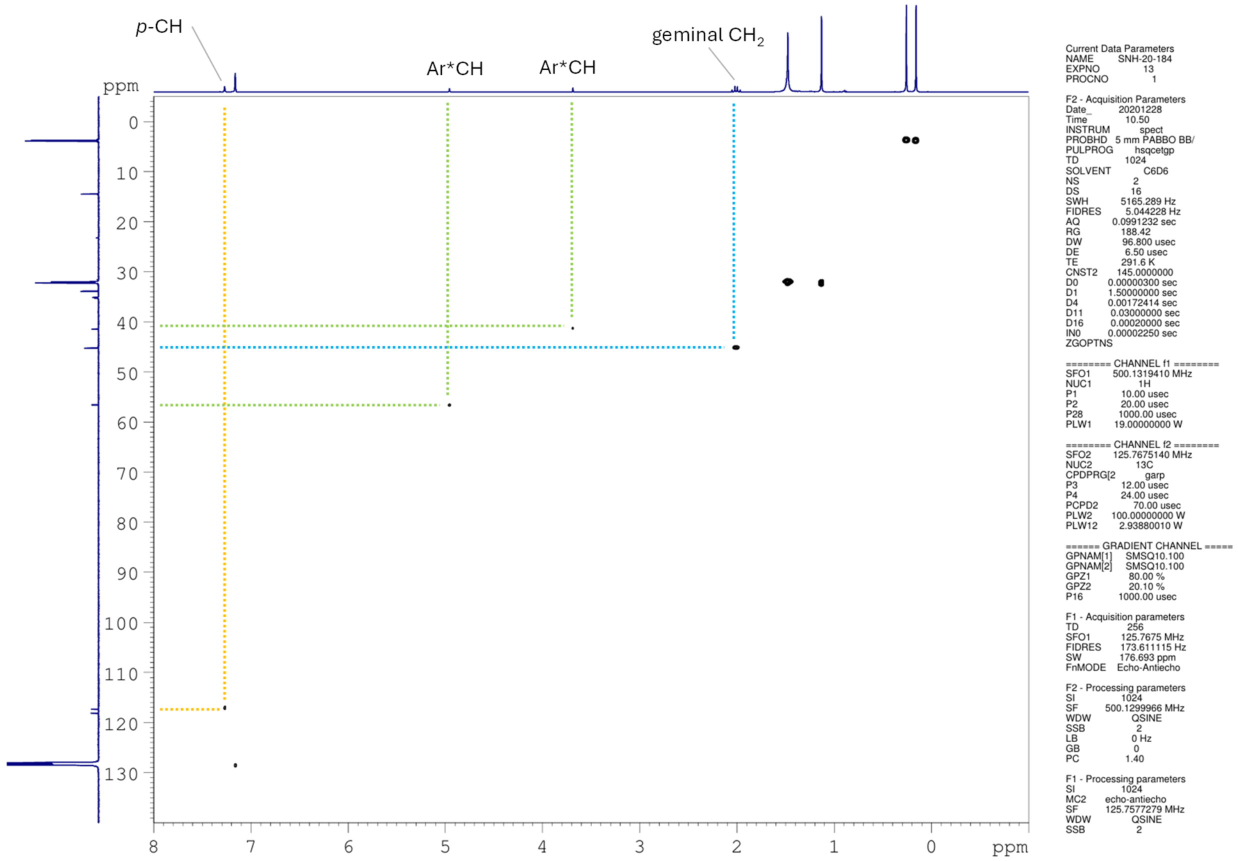

Figure S5. ^{1}H-^{13}C HSQC NMR spectrum of *trans*-**3** in C_6D_6 at 292 K.

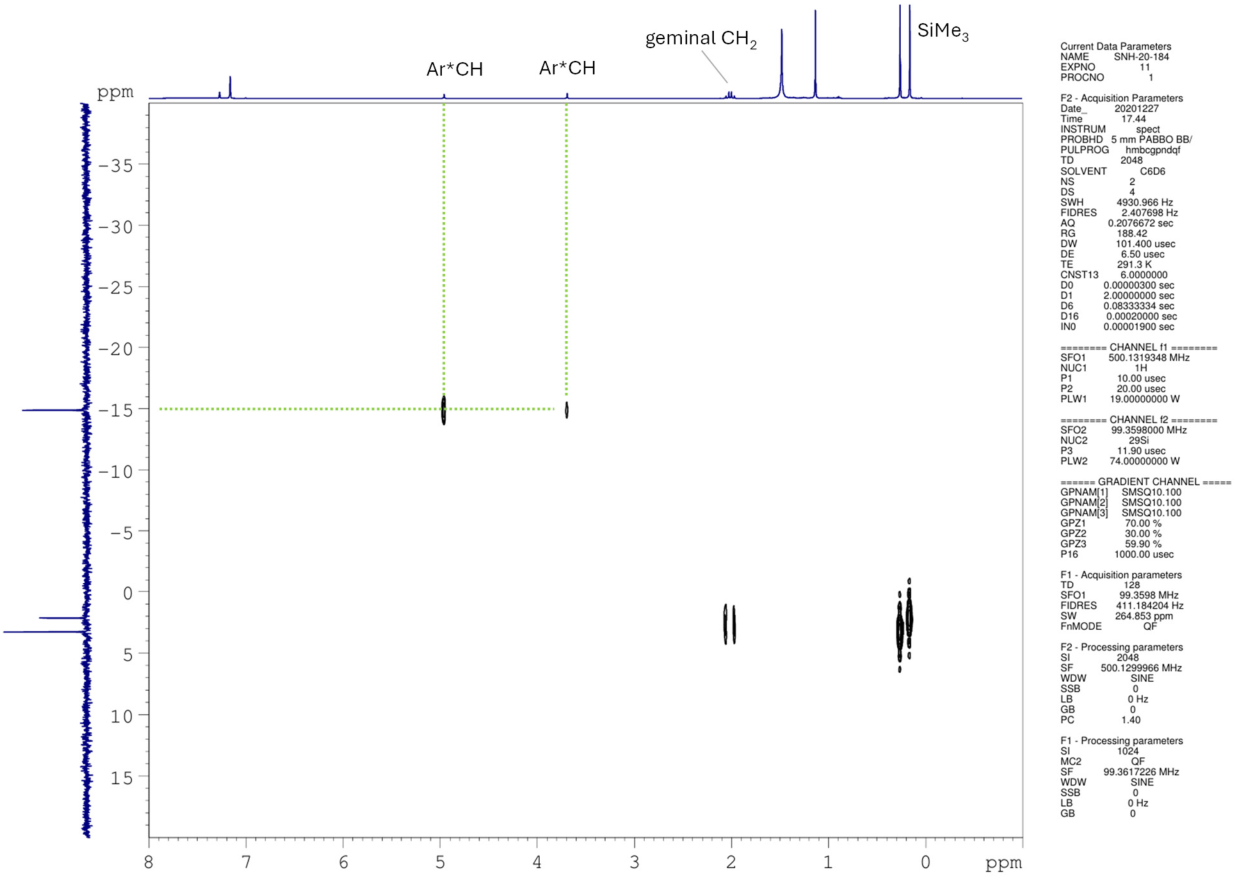

Figure S6. ^{1}H-^{29}Si HMBC NMR spectrum of *trans*-**3** in C_6D_6 at 291 K.

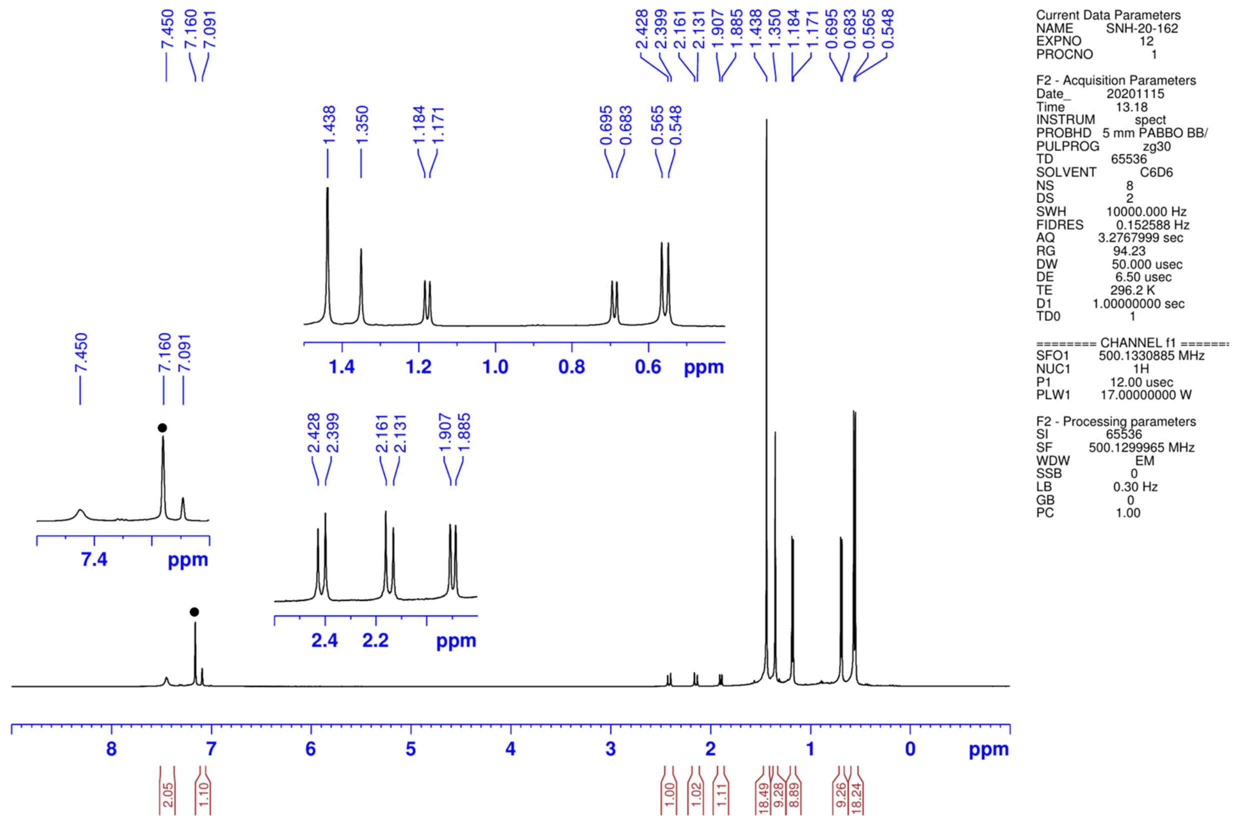

Figure S7. ^{1}H NMR spectrum of **4** in C_6D_6 at 296 K (• = C_6D_5H).

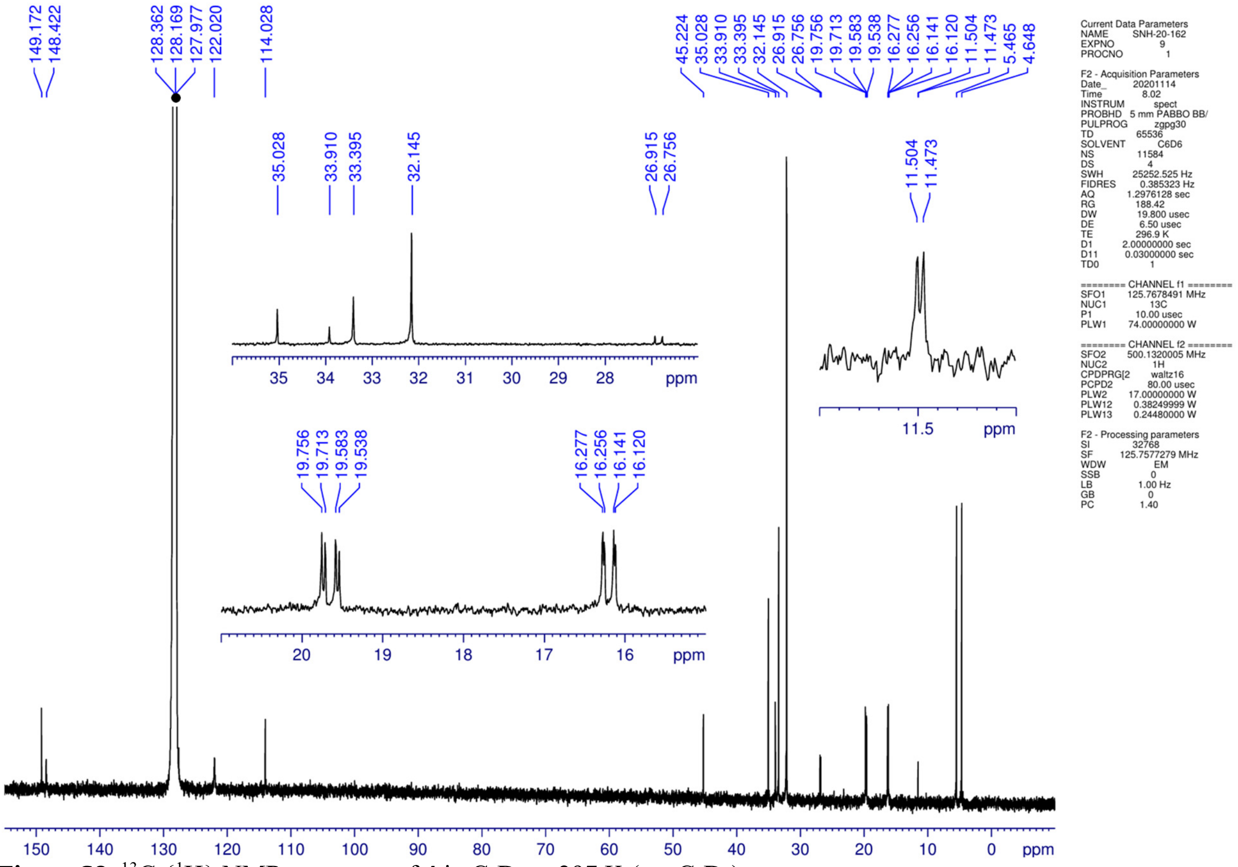

Figure S8. ^{13}C {^{1}H} NMR spectrum of **4** in C_6D_6 at 297 K (• = C_6D_6).

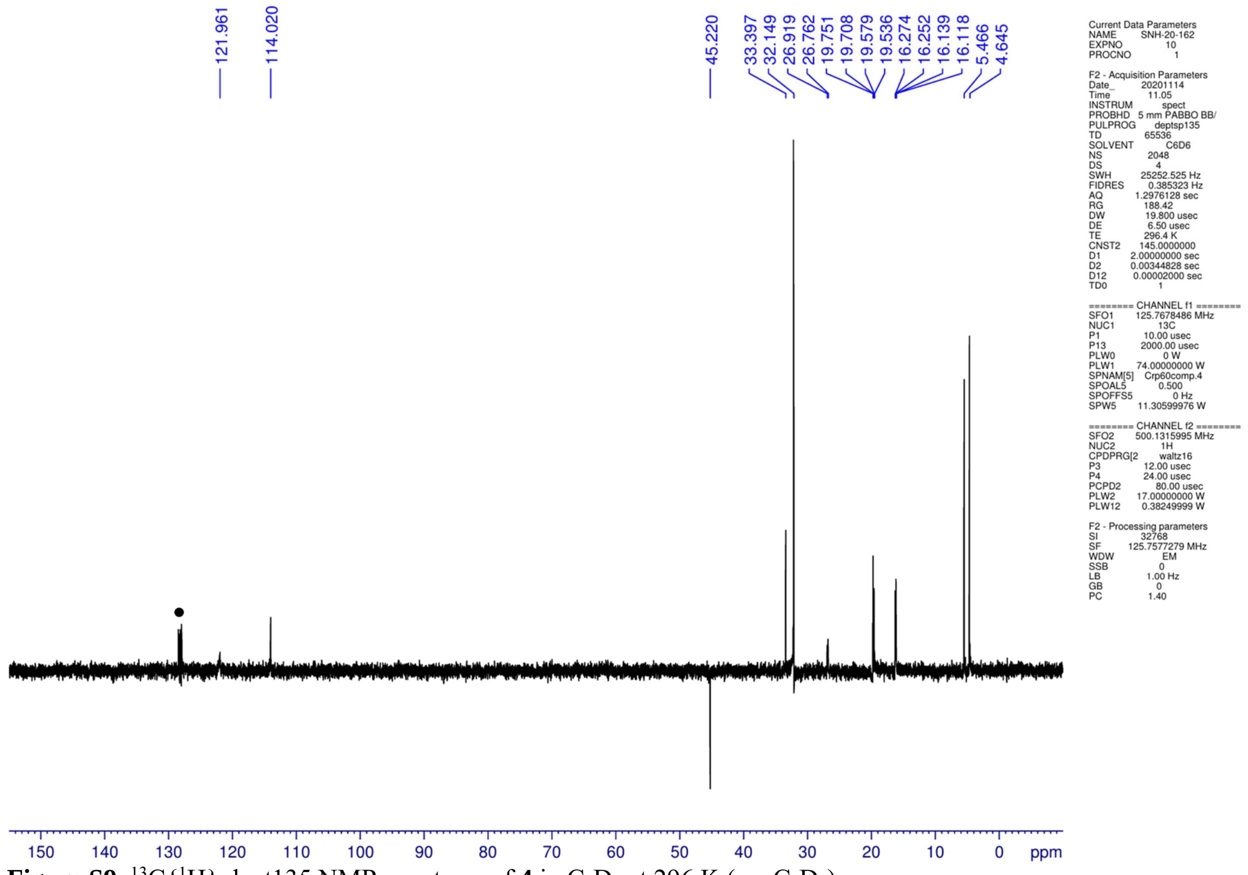

Figure S9. $^{13}C\{^1H\}$ dept135 NMR spectrum of **4** in C_6D_6 at 296 K (• = C_6D_6).

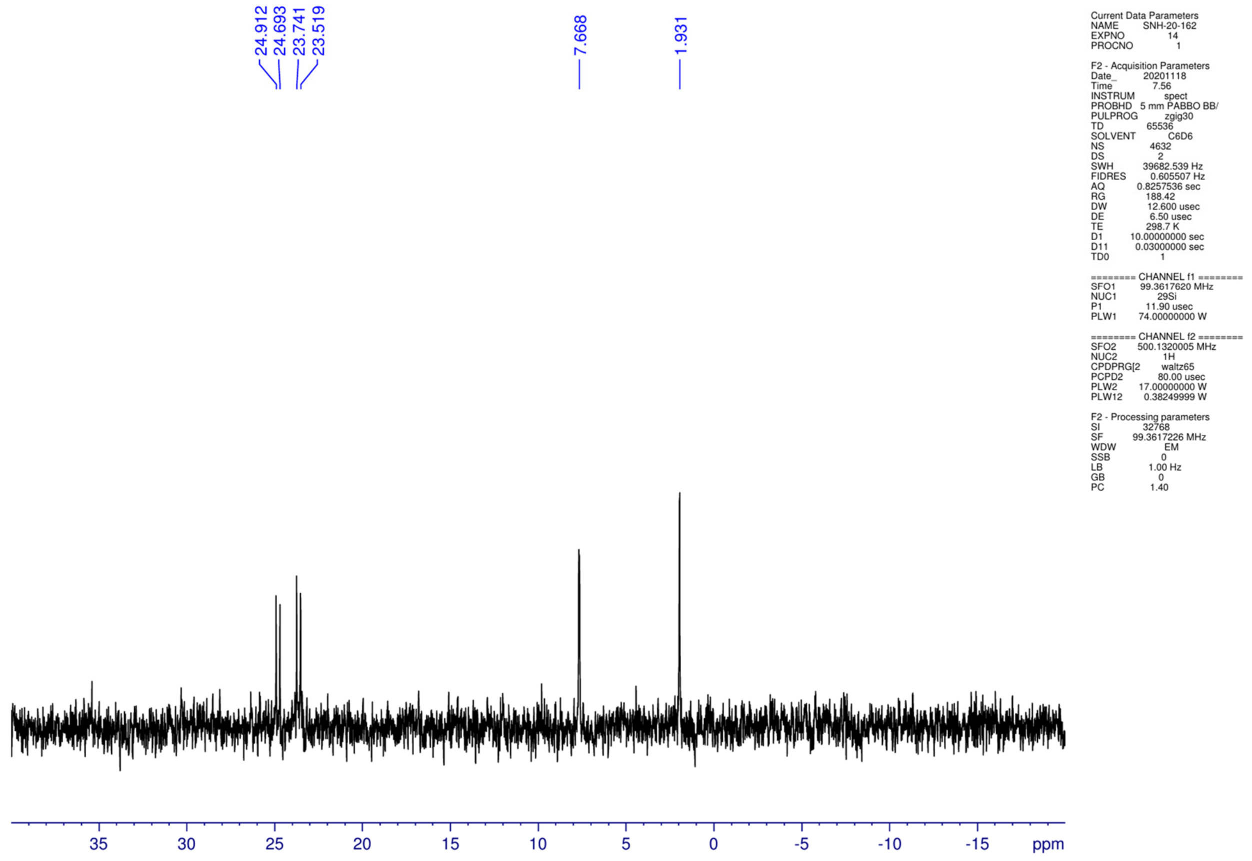

Figure S10. $^{29}Si\{^1H\}$ NMR spectrum of **4** in C_6D_6 at 299 K.

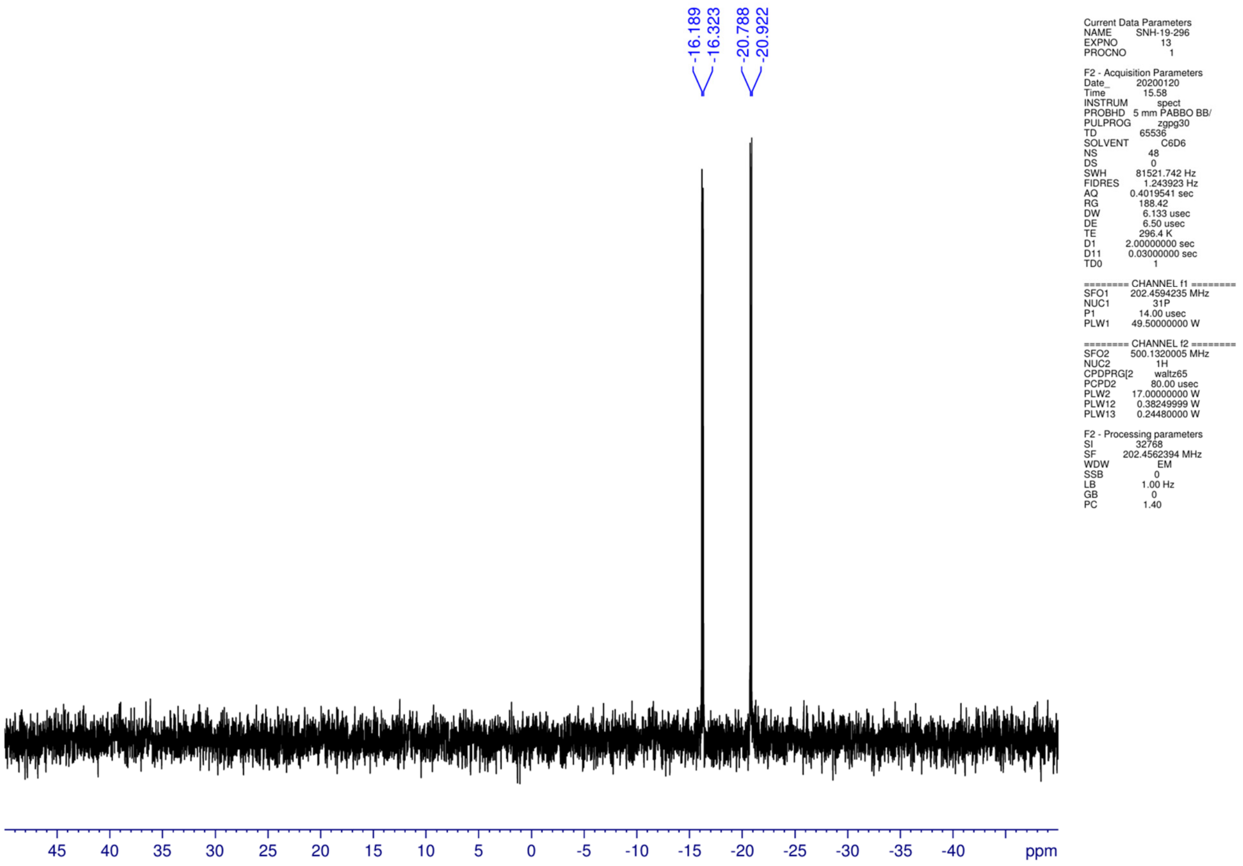

Figure S11. $^{31}P\{^{1}H\}$ NMR spectrum of **4** in C_6D_6 at 296 K.

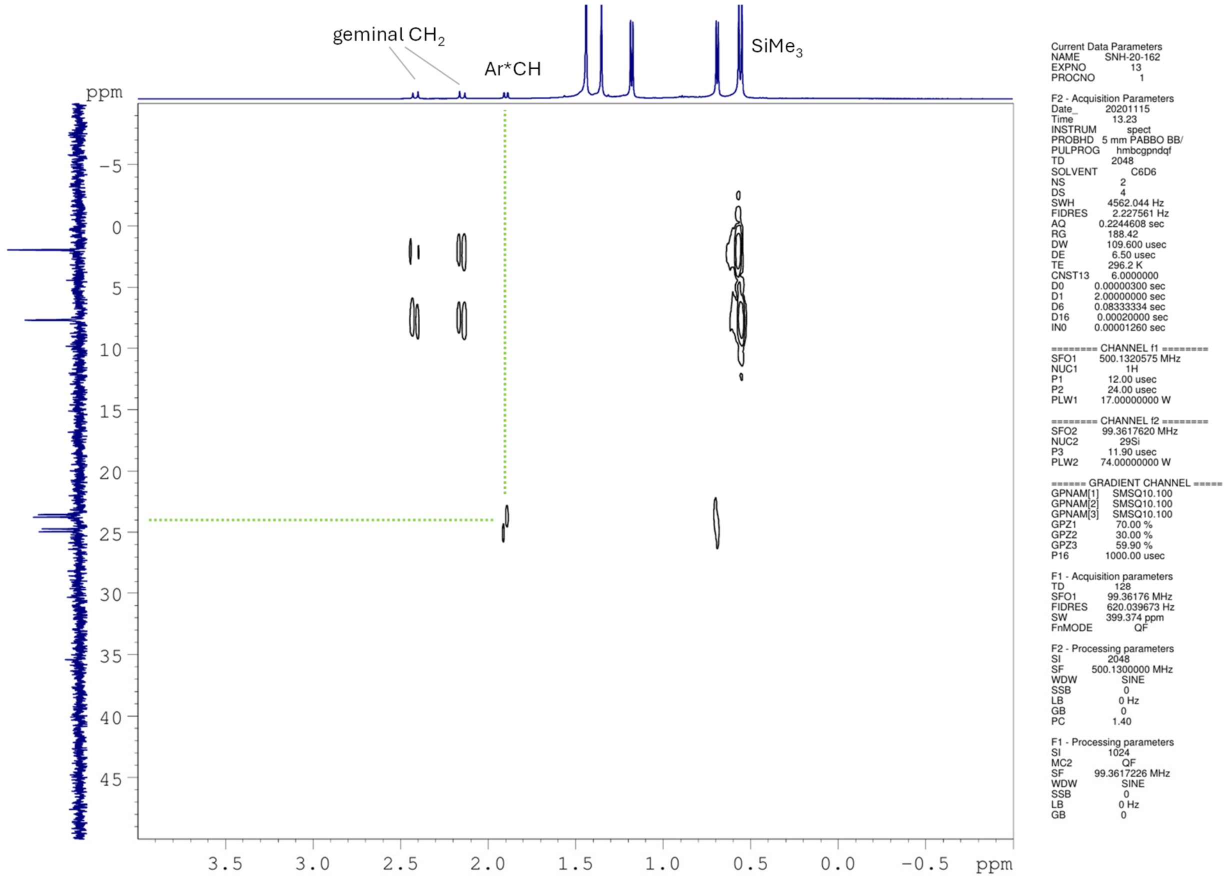

Figure S12. ^{1}H-^{29}Si HMBC NMR spectrum of **4** in C_6D_6 at 296 K.

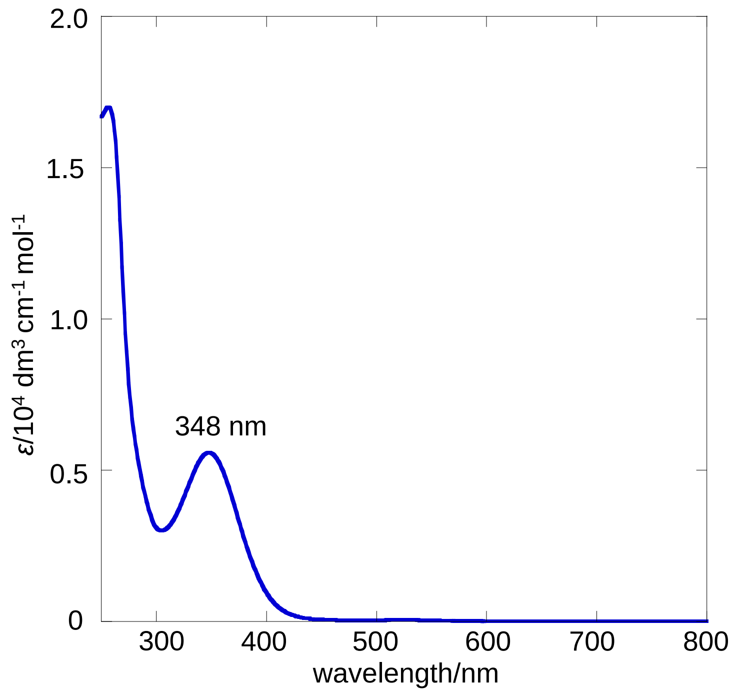

Figure S13. UV-vis spectrum of *trans*-**3** in hexane at room temperature.

2. X-ray Crystallographic Analysis Data

Table S1. Crystal data and structure refinement for bicyclobutane *trans*-**3** (CCDC 2519480)

Empirical formula	$C_{54}H_{102}Si_6$
Formula weight	919.89
Temperature	100(2) K
Crystal system	Orthorhombic
Space group	*Pnma*
Unit cell dimensions	a = 37.257(2) Å
	b = 14.8820(9) Å
	c = 10.6685(6) Å
Volume	5915.2(6) Å^3
Z	4
Density (calculated)	1.033 Mg/m^3
Absorption coefficient	0.172 mm^{-1}
F(000)	2040
Crystal size	0.100 × 0.020 × 0.020 mm^3
Theta range for data collection	1.751 to 25.374°.
Index ranges	$-44<=h<=44$, $-17<=k<=17$, $-12<=l<=12$
Reflections collected	70969
Independent reflections	5631 [R(int) = 0.1088]
Completeness to theta = 25.242°	99.8 %
Absorption correction	Empirical
Max. and min. transmission	0.7452 and 0.6883
Refinement method	Full-matrix least-squares on F^2
Data / restraints / parameters	5631 / 258 / 341
Goodness-of-fit on F^2	1.059
Final R indices [$I > 2\sigma(I)$]	$R1$ = 0.0607, $wR2$ = 0.1394
R indices (all data)	$R1$ = 0.0894, $wR2$ = 0.1565
Largest diff. peak and hole	0.895 and –0.368 e·Å^{-3}

Table S2. Crystal data and structure refinement for **4** (CCDC 2519479)

Empirical formula	$C_{33}H_{69}BrNiP_2Si_3$	
Formula weight	750.71	
Temperature	100(2) K	
Crystal system	Monoclinic	
Space group	$P2_1$	
Unit cell dimensions	a = 9.6105(15) Å	
	b = 24.281(4) Å	β = 102.978(4)°
	c = 17.984(3) Å	
Volume	4089.4(11) Å^3	
Z	4	
Density (calculated)	1.219 Mg/m^3	
Absorption coefficient	1.638 mm^{-1}	
F(000)	1608	
Crystal size	0.100 × 0.010 × 0.010 mm^3	
Theta range for data collection	1.162 to 25.471°.	
Index ranges	-11<=h<=11, -29<=k<=29, -21<=l<=21	
Reflections collected	49604	
Independent reflections	15088 [R(int) = 0.0885]	
Completeness to theta =25.242°	100.0 %	
Absorption correction	Empirical	
Max. and min. transmission	0.7452 and 0.5263	
Refinement method	Full-matrix least-squares on F^2	
Data / restraints / parameters	15088 / 1 / 795	
Goodness-of-fit on F^2	1.012	
Final R indices [$I > 2\sigma(I)$]	$R1$ = 0.0501, $wR2$ = 0.1093	
R indices (all data)	$R1$ = 0.0747, $wR2$ = 0.1213	
Largest diff. peak and hole	1.062 and –0.746 e·Å^{-3}	

3. Computational Study

Table S3. Transition Energies, Wavelength and Oscillator Strength of the Electronic Transitions of **3_{opt}**

```
 Excited State   1:      Singlet-A      3.5780 eV  346.52 nm  f=0.1929
<S**2>=0.000
     255 -> 256        0.68860

 Excited State   2:      Singlet-A      3.9259 eV  315.81 nm  f=0.0084
<S**2>=0.000
     253 -> 256       -0.17732
     254 -> 256        0.65180

 Excited State   3:      Singlet-A      4.3551 eV  284.69 nm  f=0.0138
<S**2>=0.000
     248 -> 256       -0.10623
     253 -> 256        0.62378
     254 -> 256        0.15855
     254 -> 267       -0.13899

 Excited State   4:      Singlet-A      4.4905 eV  276.10 nm  f=0.0035
<S**2>=0.000
     255 -> 261        0.54999
     255 -> 262        0.13087
     255 -> 264       -0.19561
     255 -> 267       -0.31210

 Excited State   5:      Singlet-A      4.6080 eV  269.06 nm  f=0.0091
<S**2>=0.000
     255 -> 264        0.50679
     255 -> 267       -0.42136

 Excited State   6:      Singlet-A      4.7645 eV  260.23 nm  f=0.0112
<S**2>=0.000
     255 -> 257        0.49042
     255 -> 259        0.21197
     255 -> 263        0.13551
     255 -> 265        0.10040
     255 -> 269        0.20881
     255 -> 287       -0.10342
     255 -> 288        0.23296
     255 -> 293        0.12352

 Excited State   7:      Singlet-A      4.8315 eV  256.61 nm  f=0.0027
<S**2>=0.000
     253 -> 256       -0.10264
     253 -> 267        0.13620
     254 -> 261        0.13392
     254 -> 267       -0.19458
     255 -> 257        0.11660
     255 -> 259       -0.20709
     255 -> 269       -0.11380
     255 -> 278       -0.18356
     255 -> 279       -0.11045
     255 -> 280        0.17672
     255 -> 283       -0.21076
     255 -> 284        0.26496
     255 -> 287        0.15688
     255 -> 288        0.13214
     255 -> 292       -0.10716

 Excited State   8:      Singlet-A      4.8882 eV  253.64 nm  f=0.2067
<S**2>=0.000
     251 -> 264        0.12386
     253 -> 261        0.12226
     253 -> 264       -0.11872
     253 -> 272       -0.13823
     253 -> 275        0.14953
     254 -> 261        0.42504
     254 -> 262        0.10043
     254 -> 264       -0.29400
     254 -> 267       -0.13966
     255 -> 257       -0.13232

 Excited State   9:      Singlet-A      4.9428 eV  250.84 nm  f=0.0083
<S**2>=0.000
     252 -> 261       -0.13773
     252 -> 267        0.11388
     253 -> 264       -0.13951
     253 -> 267        0.19491
     253 -> 276        0.13199
     254 -> 264        0.28061
     254 -> 267       -0.27284
     254 -> 276       -0.17639
     255 -> 257       -0.18295
     255 -> 259        0.11512
     255 -> 283        0.10488
     255 -> 284       -0.13533

 Excited State  10:      Singlet-A      5.0304 eV  246.47 nm  f=0.0066
<S**2>=0.000
     255 -> 257       -0.23153
     255 -> 259       -0.19506
     255 -> 260        0.31999
     255 -> 268        0.11833
     255 -> 269        0.15326
     255 -> 277       -0.11678
     255 -> 278       -0.13311
     255 -> 288        0.26362
     255 -> 289        0.14037
     255 -> 291       -0.13330
     255 -> 292       -0.12996
     255 -> 293        0.16934
```

```
 Excited State  11:      Singlet-A      5.1206 eV  242.13 nm  f=0.1779
<S**2>=0.000
     251 -> 261       -0.12547
     251 -> 264        0.15678
     252 -> 256        0.10616
     253 -> 261        0.21467
     253 -> 267       -0.16618
     253 -> 276        0.15658
     254 -> 261       -0.20333
     254 -> 264        0.14605
     254 -> 272       -0.21027
     254 -> 275        0.25092
     255 -> 257        0.11890
     255 -> 259       -0.15124

 Excited State  12:      Singlet-A      5.1574 eV  240.40 nm  f=0.0122
<S**2>=0.000
     255 -> 258        0.61496
     255 -> 272       -0.13079
     255 -> 276       -0.23563

 Excited State  13:      Singlet-A      5.2027 eV  238.31 nm  f=0.0927
<S**2>=0.000
     255 -> 257       -0.28771
     255 -> 259        0.44339
     255 -> 260       -0.18252
     255 -> 278       -0.11996
     255 -> 283       -0.13920
     255 -> 284        0.14002
     255 -> 288        0.18253

 Excited State  14:      Singlet-A      5.2596 eV  235.73 nm  f=0.0508
<S**2>=0.000
     252 -> 256        0.12656
     253 -> 261        0.17125
     253 -> 264       -0.20713
     254 -> 264       -0.14376
     254 -> 267        0.16875
     254 -> 270        0.10098
     254 -> 276       -0.21789
     255 -> 259        0.22472
     255 -> 260        0.30209
     255 -> 266       -0.10403
     255 -> 276       -0.10125
     255 -> 284        0.10901

 Excited State  15:      Singlet-A      5.2633 eV  235.56 nm  f=0.0053
<S**2>=0.000
     255 -> 258        0.28533
     255 -> 264       -0.14750
     255 -> 270       -0.10077
     255 -> 271        0.21304
     255 -> 272        0.25018
     255 -> 275       -0.12388
     255 -> 276        0.41861

 Excited State  16:      Singlet-A      5.2755 eV  235.02 nm  f=0.0073
<S**2>=0.000
     252 -> 256       -0.13534
     253 -> 261       -0.13158
     253 -> 264        0.15311
     254 -> 267       -0.13160
     254 -> 276        0.15655
     255 -> 259        0.18492
     255 -> 260        0.44814
     255 -> 269       -0.10894
     255 -> 288       -0.12047

 Excited State  17:      Singlet-A      5.3170 eV  233.18 nm  f=0.0008
<S**2>=0.000
     253 -> 259        0.12662
     253 -> 260        0.12171
     254 -> 257        0.59242
     254 -> 260        0.11275
     254 -> 263        0.10938
     254 -> 288        0.13821

 Excited State  18:      Singlet-A      5.3647 eV  231.11 nm  f=0.0019
<S**2>=0.000
     255 -> 262        0.22983
     255 -> 270       -0.24517
     255 -> 272       -0.31970
     255 -> 275        0.40979
     255 -> 276        0.20599

 Excited State  19:      Singlet-A      5.4455 eV  227.68 nm  f=0.0034
<S**2>=0.000
     255 -> 257       -0.13702
     255 -> 259       -0.13227
     255 -> 263        0.55581
     255 -> 265        0.30851

 Excited State  20:      Singlet-A      5.4694 eV  226.69 nm  f=0.0324
<S**2>=0.000
     253 -> 257        0.30180
     254 -> 259        0.51420
     254 -> 260        0.17443
```

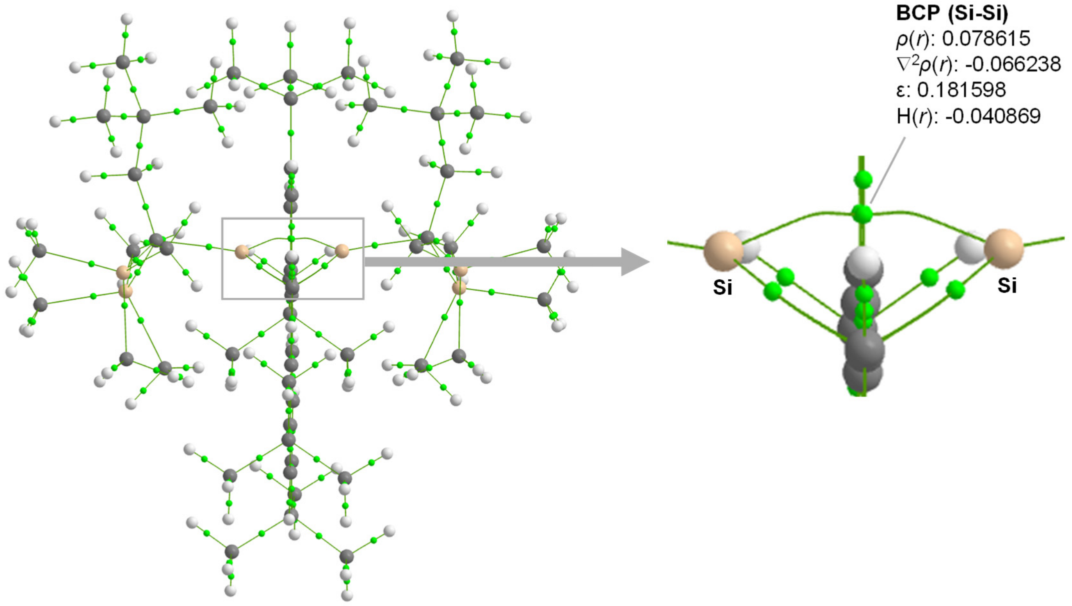

Figure S14. Molecular graph of $\mathbf{3}_{\mathbf{opt}}$. Green spheres: bond critical points (BCP), green lines: bond paths: $\rho(r)$: electron density in $e\ a_0^{-3}$; $\nabla^2\rho(r)$: Laplacian of electron density in $e\ a_0^{-5}$; ε: bond elipticity; H(r): total enegy density function in $e\ a_0^{-3}$.

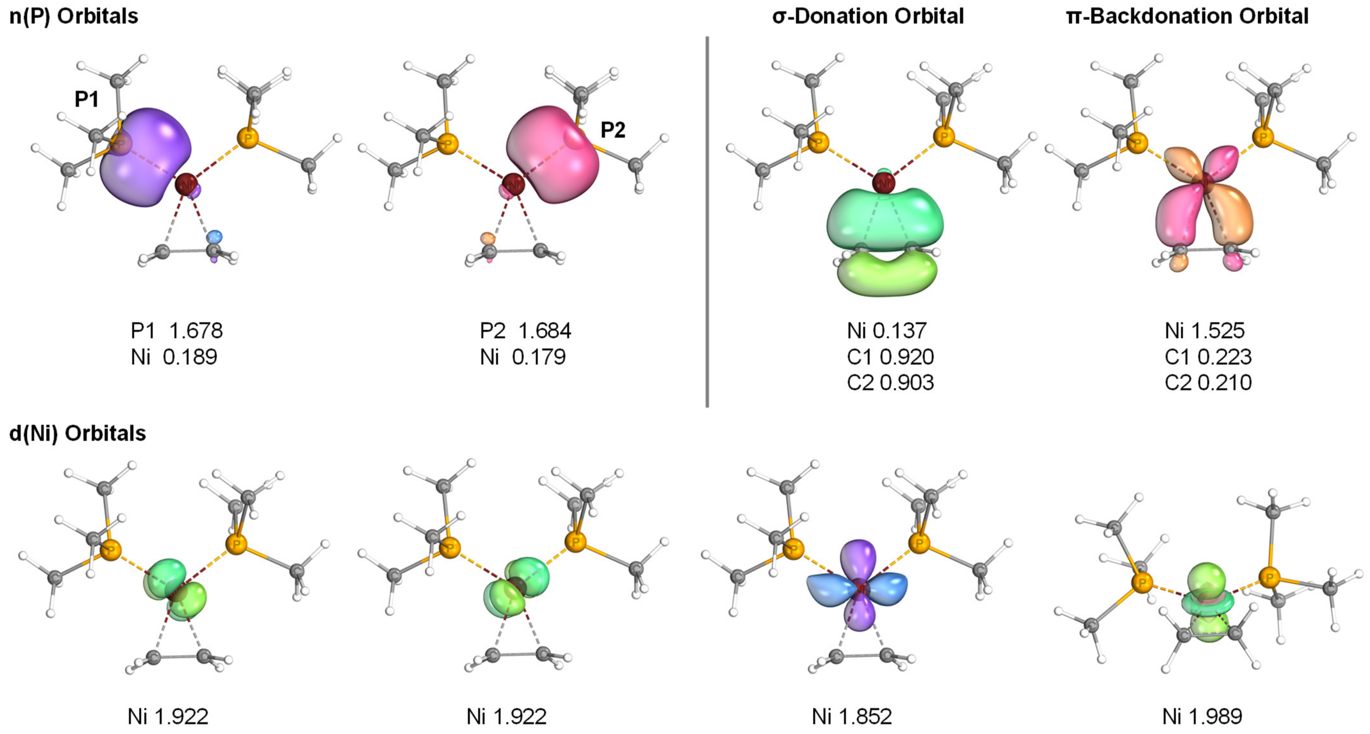

Figure S15. Selected IBOs of Ni(PMe_3)$_2$(η^2-ethylene) calculated at the PBE/def2-TZVP//M06-2X/B1 [B1 basis: SDD for Ni atom: 6-31G(d) for H, C, and P atoms] level of theory. Values indicate electron numbers on atoms in each IBO.
